# The feasibility and acceptability of a rewards system based on food purchasing behaviour in secondary school cashless canteens: the Eat4Treats (E4T) cluster feasibility, non-randomised, controlled intervention study

**DOI:** 10.1186/s40814-023-01436-6

**Published:** 2024-01-09

**Authors:** Sarah E. Moore, Ciara Rooney, Charlotte E. Neville, Ryan McConville, Frank Kee, Claire T. McEvoy, Jayne V. Woodside, Judith Hanvey, Michelle C. McKinley

**Affiliations:** 1 Centre for Public Health, Queen’s University Belfast, Institute of Clinical Science A, Grosvenor Road, Belfast, BT12 6BA UK; 2 Department of Engineering Mathematics, University of Bristol, Ada Lovelace Building, University Walk, Bristol, BS8 1TW UK; 3 Education Authority Northern Ireland, 40 Academy Street, Belfast, BT1 2NQ UK

**Keywords:** Food choice, Nutrition, School food, Diet, Adolescents, Rewards, Intervention

## Abstract

**Background:**

Using rewards may be an effective method to positively influence children’s eating behaviour but evidence to date is limited, particularly in older children. The cashless canteen systems in schools provides a unique opportunity to implement a food-based reward scheme but intervention development work and feasibility testing is needed. The overall aim of the E4T feasibility study was to examine the feasibility and acceptability of implementing a rewards scheme based on the food purchasing behaviour of pupils in cashless canteens in secondary schools.

**Methods:**

A non-randomised, controlled, parallel-group cluster feasibility study conducted in four secondary schools (two intervention and two control) serving areas of the highest social deprivation in Northern Ireland. During the 4-month trial, pupils earned points for foods purchased at the school canteen, with better nutritional choices having a higher value. Pupils could exchange the points they earned for rewards (e.g. stationery, vouchers, sports equipment) via the E4T website. Qualitative and quantitative data was collected from year 9 and 10 pupils (boys and girls aged 12–14 years), teachers and canteen staff to address the feasibility questions.

**Results:**

Two intervention (one urban, one rural) and one control (urban) school completed the study. Seventy-one percent of 12–14-year-old pupils consented to take part; 1% of parents opted their child out of the study. Questionnaire completion rates were high (6 and 11% of questionnaires were partially completed at baseline and follow-up respectively). Collecting data on food consumed in the canteen was challenging logistically. Focus groups with pupils indicated that the overall concept of E4T was well received and there was a high degree of satisfaction with the rewards available. Pupils and teachers made several suggestions for improvements.

**Conclusions:**

E4T was successfully implemented as a result of collaboration between schools, school canteens and cashless canteen providers working with a multidisciplinary research team. It was acceptable to pupils, teachers and canteen staff. The findings suggest a few areas for refining implementation and evaluation processes that would need to be considered in the design of a larger trial, particularly resources required to streamline implementation and ways to optimise pupil engagement.

**Trial registration:**

Under review with https://www.clinicaltrials.gov (retrospective registration—reg number and weblink to be added).

**Supplementary Information:**

The online version contains supplementary material available at 10.1186/s40814-023-01436-6.

## Key messages regarding feasibility

- *What uncertainties existed regarding the feasibility?*

The aim of the E4T feasibility study was to examine the feasibility and acceptability of implementing a rewards scheme based on the food purchasing behaviour of pupils in cashless canteens in secondary schools.

- *What are the key feasibility findings?*

The E4T scheme was acceptable and was feasible to implement. A considerable resource was required to prepare the cashless canteen data capture and train canteen staff ready for implementation of the E4T scheme.

- *What are the implications of the feasibility findings for the design of the future trial?*

The intervention was well received by pupils and school staff. Future studies can use the findings from this study to refine the features and delivery of a rewards-based intervention targeting dietary behaviour in secondary school pupils. This work also provides valuable information on conducting school-based health research and working with existing technology in schools to implement behaviour change interventions.

## Background

The diet of UK children is sub-optimal, particularly with regard to higher than recommended intakes of saturated fat and sugar, and low intakes of fibre and fruit and vegetables, in a large proportion of children [1]. The Health Survey for England [2] reported that 18% of children aged 5 to 15 years old ate the recommended five or more portions of fruit and vegetables per day and only 8% of children aged 11–18 years old met the recommendation according to the UK National Diet and Nutrition Survey [1]. Notably, a substantial proportion of children aged 11–18 years old have low intakes (below the UK Lower Reference Nutrient Intake) of a number of micronutrients including vitamin A, vitamin B2, calcium, iron (in girls), magnesium, potassium, iodine, selenium and zinc [1].

Inequalities in accessing a healthy diet contribute to poor health. In 2019–2020, 31% of children in the UK were reported to be living in poverty, equating to nine children in a classroom of 30 pupils [3, 4]. There is a need to develop effective and sustainable ways of helping young people access and choose a better diet, particularly those from more disadvantaged backgrounds. Schools can play a crucial role in addressing inequalities and improving the health of *all* children. Children spend approximately 40% of their waking hours in the school environment and consume, as a minimum, break and lunch in this environment. Thus, school food is primed to make a substantial contribution to a child’s daily intake of energy, fat, fibre and other nutrients.

There has been a significant improvement in school meal provision throughout the UK in recent years following the implementation of statutory nutritional standards. However, the improved nutritional profile of school meals does not automatically mean that children will make the best nutritional choices in the canteen. Often students still choose a limited variety of foods in school [5, 6] and school meal uptake decreases as children move through secondary school; 43% of 11 year olds versus 36% of 15 year olds usually have a school meal in Northern Ireland [7]. Furthermore, uptake of free school meal entitlement is sub-optimal; for example, the Scotland School Healthy Living Survey, carried out in all publicly funded schools annually, found 76% of those registered for free school meals and present on the survey day in 2020 [8]. Similarly, for Northern Ireland (which is part of the UK), the uptake of free school meals by entitled pupils was 75% in 2020/21 [9]. Secondary school canteens also face significant competition from external food providers in close proximity to the school grounds, particularly for older pupils.

In relation to school lunch-time, an important development in schools is the increasing implementation of cashless canteen systems (CCS). CCS are now widely used in secondary schools in the UK, particularly as schools move towards a cash-free environment for all school activities [10]. These systems can provide a full transaction history for every child using them, thus providing an opportunity to monitor the food purchasing behaviour of young people. Schools and the purveyors of these systems [11] report significant benefits for both pupils and catering staff, including increasing uptake of free school meals, reduced bullying and theft, reduced queuing time and better stock management.

The CCS in schools also provide a unique opportunity to promote a varied and balanced diet through appropriate marketing and health promotion activities, with one example being the use of reward schemes to encourage pupils to eat in the school canteen and to promote healthier eating practices. Offering rewards or positive reinforcement for healthy dietary changes is proposed to establish extrinsic motivation to continue performing the behaviour, subsequently increasing the likelihood of the behaviour becoming habitual [11]. A 2004 Food Standards Agency report based on 79 schools from England (5695 pupils age 11–18 years), reported that five schools attempted to use ‘smart-card’ CCS to encourage healthier choices by awarding points which could be exchanged for vouchers or gifts. When the smart cards were linked to reward points for making healthy choices, pupils chose soft drinks less often (13% *vs.* 16%), and chose low-fat starches (rice, pasta, bread, potatoes not cooked in oil) (8% *vs.* 5%), baked beans (6% *vs.* 4%) and low-fat main meals (9% *vs.* 6%) more often. There was no effect, however, on the percent choosing chips or high-fat main meals [12]. On a larger scale, in 2004, Glasgow City Council devised a website for a point-based healthy eating CCS reward scheme called ‘Fuel Zone’ which was rolled out to 29 secondary schools. The ‘Fuel Zone’ concept also involved school dining hall refurbishments (between 1996 and 1999) and an increase in healthy options available for purchase (between 2002 and 2004). An evaluation in 2006 reported that over 800 rewards were issued between January and June 2006, school meal update in secondary schools increased from 32% pre-Fuel Zone to 60% and consumption of balanced meals increased from 30% pre-Fuel Zone to 60% [13]. A later evaluation in 2010 reported that registration for the reward scheme remained steady at approximately 25% of Glasgow pupils and about 4200 rewards were distributed to pupils between 2007 and 2010 as a result of making healthier food choices. [13]

Peer-reviewed literature to date has shown some promising findings regarding the effectiveness of rewards to improve dietary behaviour in children in the short term [14–16]. Existing studies have largely focused on whether rewards can increase fruit and vegetable intake among primary school aged children. The ‘Kids Choice’ [15] and ‘Food Dudes’ [16] interventions both used rewards to encourage fruit and vegetable consumption in primary school children; both reported increased consumption of fruit and vegetables in school, and also in the home setting for the latter intervention [16]. In general, however, peer-reviewed evidence regarding the effectiveness of using rewards to encourage dietary behaviour change in children is very sparse, is limited to children under 11 years old and requires further research. The CCS that are widely used in secondary schools offers an ideal opportunity to implement a rewards-based intervention; however, development work and feasibility testing is needed to inform the viability of this approach.

The overall aim of the E4T feasibility study was to examine the feasibility and acceptability of implementing a rewards scheme based on the food purchasing behaviour of pupils in cashless canteens in secondary schools focusing on schools serving the most disadvantaged areas (based on postcode data for individual pupils attending the school) and pupils aged 12–14 years old.

The specific feasibility study objectives addressed in this manuscript were to:

1. Evaluate the recruitment of schools and consent processes for data collection;
2. Evaluate the appropriateness of the data collection procedures including acceptability to pupils aged 12–14 years old and staff involved in implementing the intervention;
3. Evaluate the acceptability of the E4T scheme to pupils aged 12–14 years old and stakeholders involved in implementing the intervention;
4. Evaluate resources needed to implement the E4T scheme and collect outcome data.

[Note—examination of whether the intervention shows promise with regard to influencing the food purchasing habits of pupils will be reported separately.]

## Methods

### Development of the E4T intervention

The concept of the E4T intervention came from discussions with members of the Northern Ireland Food in Schools forum (which supports the implementation of the Food in Schools policy and includes representatives from the Department of Education, Department of Health, and the Public Health Agency) who wanted to increase the proportion of pupils using the school canteen, increase uptake of free school meals, and, overall, encourage pupils to make the best choices in the canteen.

The development of the E4T intervention is described below and Additional file 1 details the behaviour change techniques (BCTs) that were incorporated. The intervention was underpinned by socio-cognitive theories of behaviour change. Socio-cognitive models of behaviour rely on the balance of positive and negative consequences of performing a given behaviour as judged by the individual and attempt to induce behaviour change by altering an individual’s judgements via increasing awareness, increasing motivation, and increasing self-efficacy [17]. The rewards-based intervention intended to increase healthy food consumption through increasing awareness of relevant behaviours, but also by increasing motivation and self-efficacy, through goal-setting, self-monitoring and social comparison. The receipt of points based on food choices was intended to make students aware of their behaviour alongside encouragement to set food choice goals, and to self-monitor and review their goals on a regular basis. Goal-setting, rewards for goal achievement and rewards for target behaviours have all previously been successfully used to increase health behaviours; a meta-regression of effective techniques in healthy eating and physical activity interventions found that interventions using self-monitoring combined with at least one other technique were significantly more effective than other interventions [18]. The concept of a rewards-based intervention associated with food choice in school canteens was explored in focus groups with pupils in 10 schools (90 pupils aged 11–12 years (54 girls, 36 boys) located in lower socioeconomic status areas within Northern Ireland [19]. Our findings indicated a high degree of acceptability for a reward scheme, but there was diversity in the type of rewards valued by pupils, largely defined by geographical area and socio-cultural differences which were factored into the rewards offered in the E4T intervention. Pupils from rural areas tended to emphasise group-based and longer-term rewards, whereas pupils from urban-city schools tended to suggest individualistic and immediate rewards [19].

### Overview of the E4T intervention

E4T is a food-based reward scheme developed for implementation in school canteens that operate a CCS (i.e. a system that allows purchases to be made using an alternative means to cash, e.g. fingerprint, card). CCS require catering staff to key in food and beverage selections on a touchscreen till and enable a full transaction history (i.e. pupil code, food item, time and date of purchase, food cost and price paid) to be obtained for every pupil.

The E4T intervention consisted of a website (was also available as a mobile application for Android phones but was not available on iOS owing to budget limitations), large posters and leaflets advertising the scheme, a booklet to support canteen staff with the implementation of E4T, an ‘education pack’ for teachers and an initial information session about the scheme delivered by researchers.

Rewards were contingent on the target behaviour, i.e. students received ‘points’ based on food choices, with better nutritional choices having a higher point value. Rewards were selected based on our previous qualitative research with pupils [19] and based on discussion with the recruited intervention schools. To cater for a range of preferences, rewards included a range of lower value items, such as stationery, to higher-value items, such as headphones. For the feasibility study, when pupils claimed rewards via the website, they were purchased by a study researcher and posted to the intervention schools for distribution to pupils by school staff.

Pupils registered to take part in E4T via the E4T website in a two-step process: Step 1—pupils submitted their name, class, and school email address; Step 2—an email was automatically sent to their school email address which pupils used to confirm their identity and complete registration. Supplying this information enabled pupil’s accounts on the E4T website to be linked with their purchasing information collected in the school canteen. Through the E4T website, pupils could monitor and exchange the points they earned for non-food rewards of varying monetary value (see Additional file 2 for images of the different sections of the website).

### Pre-launch/preparatory work

**Fig. 1:**
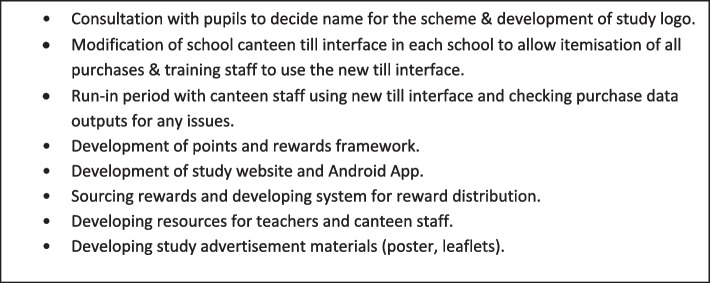
Preparatory work for E4T launch

Preparatory work required to set up E4T prior to its launch in schools took place between May 2013 and September 2015 and is summarised Fig. 1.

#### Consultation with pupils to decide name for the scheme

The scheme name was developed through an iterative process with pupils; one class of year 9 (aged 12–13 years old) pupils from two schools were presented with a list of nine potential names and asked to rate each name according to a 5-point scale: ‘I really like this name’; ‘Just OK’; ‘Neither like or dislike’; ‘Dislike’; ‘Really dislike this name’. Results were tallied to identify the name with the most favourable rating. The study logo was developed by the design team at the Public Health Agency NI who provided funding to support the intervention development and feasibility study.

#### Itemisation of canteen till interface

A large proportion of the preparation for E4T involved study researchers working with canteen supervisors in individual schools, as well as the CCS supplier to ensure the required level of detail on food purchased was captured. Initial review of the itemised purchasing data from the school CCS indicated the data captured at the point-of-sale was very generic, e.g. the output may have simply stated ‘meal of the day’, ‘dessert’ or ‘miscellaneous’. In order to implement the rewards framework which assigned points to individual foods and beverages, and needed to record specific information about the types of foods/drinks purchased, modifications to the till interface were required. For example, the button ‘Meal of the day’ was changed to a button showing the specific meal (e.g. chicken curry with rice) being purchased. Canteen menus vary between schools in NI and hence this work was done on a school-by-school basis. Researchers worked closely with canteen supervisors to devise ‘new’ screen interfaces taking into consideration the need to ensure modifications would not have a negative impact on canteen operation (e.g. queue times) and that canteen staff preferences on layout of buttons on till screens were taken into account. An example till interface at one school after modifications were made and example food purchasing outputs for one school captured before and after till modifications were made are shown in Additional file 3. Before changeover to the new till interfaces, a staff training session was held to demonstrate the changes to staff and allow them to familiarise themselves with the layout. The new till layouts were implemented for several weeks before commencement of the E4T scheme to allow changes time to embed, trouble shoot any issues arising and check that purchasing data capture was improved.

#### Point framework

In order to develop the point framework for the scheme, a list of foods and beverages offered within each of the participating schools was obtained and reviewed. Foods and beverages were then broadly categorised into ‘main meal options at lunch-time’, ‘sides’, ‘sandwiches’, ‘drinks’ and ‘desserts’. Points were assigned for each category based on a number of factors: current food purchasing habits, UK healthy eating guidelines [20] and the relative healthiness of choices within each category; Table 1 below provides an example of this for the main meal category and the full point framework can be found in Additional file 4. Every item on the menu was assigned a point value in-line with the ethos of encouraging balance and variety and encouraging pupils to have a complete meal rather than more snack-like options. The point framework was tested on a variety of food and drink combinations for sense checking. In order to achieve the top prize (headphones worth approximately £100), the rewards framework required pupils to purchase the most healthy breakfast and lunch options on most days of the intervention (school holidays and 2 days for absence were taken into consideration). Conversely, the lowest prize (stationery items) required pupils to make healthier purchases consistently for 2 weeks.

**Table 1:** Rationale for point assignment within ‘main meal’ category

Ranking (highest points to lowest points)	Points	Rationale
‘Balanced’ dishes, e.g.: Spaghetti/pasta Bolognese Vegetable curry and rice Chicken pie (potato top) Chicken curry with rice and naan bread Chicken and vegetable stir fry with noodles Salad box with meat/vegetarian Savoury fish pie Chicken jambalaya with rice Irish stew/beef stew Lasagne/vegetable lasagne	100	These options are assigned the highest points to encourage pupils to eat balanced meals at lunch (i.e. meals that consist of meat, starchy carbohydrate, vegetables and in some cases dairy). Preliminary analysis of purchasing data showed that such meals, with the exception of the meal deal and chicken curry, were not currently popular choices among pupils in one of the participating schools
Less healthy versions of the above dishes, e.g.: Chicken curry with chips and naan bread Chicken curry (half rice, half chips); pastry topped pies, creamy pasta dishes Meat on its own (with no sauces), e.g.: Roast beef/chicken/turkey Pork chop Braised steak Fillet of fish BBQ chicken drumsticks	60	Scored lower to encourage pupils to choose healthier meals (those in the category above) which are mostly lower in fat than the options in this category Meat on its own scored in this category to allow for the addition of sides to make a balanced meal
Processed meat dishes, e.g.: Sausage and bean hotpot Ham and potato bake Quiche – cheese and ham (all quiche)	50	Dietary guidelines recommend lower intake of processed meats
Pizzas (any variety)	20	Preliminary examination of purchasing data showed high consumption of pizza in participating schools—lower points allocated to encourage consumption of healthier, more balanced options
Processed meats on their own (no side), e.g.: - Processed meats / meat dishes, e.g. bacon, sausage, ham, chicken burger, hot dog, beefburger	10	See above

#### Study website and app

An E4T website was developed as the hub of the scheme which linked pupil accounts to the CCS. It allowed pupils to register for the E4T scheme, view their purchases in the school canteen, monitor their points and claim rewards, set themselves weekly goals and review them. It also provided information about how the scheme works, the points allocated to each item on the canteen menu along with the reasoning for this and healthy eating guidelines and used message boxes to provide tips, run polls and quizzes or allow pupils to suggest new rewards. Images of the study website are shown in Additional file 2.

#### Other supporting materials

In addition to the website and app, large posters and leaflets advertising the scheme, displaying the point framework and rewards available were developed and displayed in the school canteens of participating schools.

A booklet was developed to support canteen staff with the implementation of E4T, including an overview of E4T, how it would work in the canteen, some tips for increasing the success of the scheme (e.g. avoiding using unidentifiable buttons such as a ‘miscellaneous’ button where possible), and advice and contact details if any problems occurred. An ‘education pack’ was also developed for teachers, with advice from the Home Economics advisor from the Council for the Curriculum, Examinations and Assessment Northern Ireland. The pack provided two optional lesson plans (with resource sheets) on eating a balanced diet and making healthy choices in the school canteen in an aim to reinforce the key messages of E4T within the classroom.

An information session was held in each school to introduce the reward scheme to pupils.

### Study design and sample

E4T was implemented in the form of a non-randomised, controlled, cluster feasibility study. Four secondary schools in Northern Ireland (NI) that operated a CCS and were classified as ‘extended’ schools (i.e. the most disadvantaged schools according to the areas in which their pupils live, based on postcode data for individual pupils attending the school) [21] were recruited by the NI Regional Food in Schools Co-ordinator. Four schools were recruited to allow the intervention to be tested in different school canteens while also being realistic from a staff resource perspective; two (one rural, one urban) intervention schools, and two (one rural, one urban) control schools. One intervention school (urban) and one control school (urban) were single gender schools, male and female respectively. E4T ran for approximately 4 months in each school (end of September/October 2015–end of January 2016). Due to issues encountered during the sign-up process (described later within the paper), one intervention school (urban) did not complete the sign-up process until November 2015.

Information leaflets and consent forms were distributed via teachers (the form tutors) to year 9 and year 10 (aged 12–14 years old) pupils (opt-in consent) and opt-out consent forms were posted to parents. This age group was chosen as the target audience as children of this age are beginning to take more control of their food selection but are not yet completely autonomous and may benefit from guidance and education about how to look after their own diet as their level of autonomy steadily increases (this age group is not permitted to leave the school grounds at lunch-time). Schools were given a £200 voucher for a retailer of their choice and canteen staff at each school were given a £250 voucher for a retailer/local restaurant of their choice as a thank you for their participation.

### Feasibility study measures and processes (relevant objectives given in brackets)

#### Study log (objectives 1–4)

A study narrative was kept by researchers detailing processes, difficulties and challenges regarding development and implementation of the intervention and evaluation of its outcomes. This included a record of the researcher time (not including time of other co-authors) required to implement the intervention in schools and collect study data.

#### Recruitment, uptake and engagement (objectives 1 and 3)

Data recorded on study recruitment included the number of schools that took part in E4T as well as the number of pupils that provided consent for the study and the number of pupils that registered for the E4T scheme. E4T website usage statistics were used to monitor uptake (proportion of pupils registering on the E4T website) and engagement (number of visits to the website, usage of individual sections of the website, rewards claimed).

#### Research measures (objective 2)

##### Questionnaires

Pupils were asked to complete a questionnaire booklet pre- and post-participation in the E4T scheme, which examined dietary knowledge, health-related quality of life, self-efficacy, habit strength, outcome expectations, motivation, social support, social network, dietary behaviours, economic attitudes and a pupil evaluation of the scheme. Questionnaires were piloted with a sample of 18 pupils before use in the feasibility study. Schools were asked to organise designated sessions to complete the questionnaires and were given the option to complete them on paper or using an online survey platform. Researchers were not present during questionnaire completion. Completion rates and extent of missing data are reported here.

##### Assessing dietary behaviour

(i) Food purchasing behaviour was obtained from all schools via the CCS records, which provide transaction histories for individual pupils.
(ii) Overall dietary intake—the feasibility of collecting data on overall dietary intake including consumption of foodstuffs outside the school setting was examined in a sub-sample (one class) of children from each school through completion of two online 24-h dietary recalls (using INTAKE24) [22] pre- and post-implementation of the E4T scheme. Classes were selected by the school based on timetable availability. At each time point, schools were asked to facilitate pupil completion of a 24-h recall on a weekday to reflect weekday intake, and on a Monday to reflect weekend intake. At least one researcher was present during completion, with a second researcher present when possible.
(iii) Canteen observations (food waste)—A method of collecting data on food waste was included in order to examine if the intervention had any undesirable effect on this outcome, for example, children may be tempted to purchase fruit and vegetables in order to collect ‘points’ but may not consume these foods after purchase. Canteen observations were conducted to determine type of food consumed or not consumed in each of the participating schools pre- and post-intervention. One class of pupils from each school was selected by the school to take part in the canteen observation. The Digital Photography of Foods Method was employed [23, 24]. Two researchers attended the school to conduct the observation process. As each pupil entered the canteen, they were given a unique ID sticker to place on their blazer and also on their tray. After the pupil purchased their meal, they were asked to bring their plate to a photography table (pre-photos). The meals were photographed by one of the researchers using a digital camera (Nikon S700) mounted on a tripod. To standardise the images, a gridded placemat (2 × 2 cm) was used. After eating, pupils were instructed to leave their plates on their labelled tray (post-photos) at the photography table. Canteen staff were asked to provide reference portions of meals for photographs to aid with the analysis of the images.

#### Acceptability of E4T (objectives 2 and 3)

Acceptability of the research methods, data collection procedures and E4T scheme in general was assessed qualitatively among pupils in focus groups, and with principals or teaching staff and canteen supervisors who had been involved in the scheme via one-to-one interviews. The study researcher approached principals and canteen supervisors within the intervention schools to ask if they would be willing to be interviewed about their opinions on E4T. Focus group participants were selected by choosing classes with timetable availability and then selecting pupils within the class who had consented to participate in the E4T study.

The interviews and focus groups were conducted and moderated by CR. For consistency, semi-structured topic guides were devised which encompassed questions on the components of the E4T scheme (e.g. the name, the sign-up process, the rewards). Sample questions from the focus group and interview topic guides are provided in Additional file 5. Both principal and teacher interviews lasted approximately 10–20 min and canteen supervisor interviews lasted approximately 30–50 min. Focus groups lasted approximately 30 min. Interviews and focus groups were digitally recorded and transcribed verbatim. Recordings were destroyed after transcripts were prepared and checked against the recording.

#### Data analysis

Quantitative data are presented using descriptive statistics (frequencies and percentages with 95% confidence intervals).

Focus groups and interviews were transcribed verbatim by SM and CR. Data were analysed using framework analysis which is appropriate for research that has specific questions, a pre-designed sample and a priori issues to deal with [25]. SM and CR read transcripts to check accuracy and to familiarise themselves with the data. Themes were derived from a priori issues addressed in the interview schedules as well as additional themes which emerged from data. SM and CR agreed an initial coding framework then independently applied this to the transcripts using NVivo software (QSR NVivo 10). The two researchers then discussed the independently coded textual data on NVivo and agreed on a final coded dataset.

The data are presented as anonymised quotes relevant to each objective. Pupil quotes are reported as male (M) or female (F) followed by focus group number, e.g. (F, FG1).

## Results

### Evaluate the recruitment of schools and consent processes for data collection

Four secondary schools were recruited: two intervention (one urban, one rural) and two control (one urban, one rural). The rural control school withdrew prior to collection of baseline data. Pre-intervention set-up (including changes to the cashless canteen till interface to enable capture of food purchasing data) had been completed at the time of withdrawal. Reasons given by the principal for withdrawal were a lack of time to facilitate data collection and dissatisfaction with acting as a control.

Participating schools included a co-education school with a total of 453 pupils attending (rural intervention school), an all-boys school with a total of 968 pupils attending (urban intervention school) and an all-girls school with a total of 704 pupils attending (control school). Data collection focused on year 9 and 10 pupils.

Table 2 shows the study consent rates for pupils and parents. A total of 481 out of 674 (71.4%) year 9 and 10 pupils consented to take part. Only a small number of parents opted out of the study (*n* = 7; 1.0%).

**Table 2:** Proportion of pupils and parents providing consent* for the E4T feasibility study

	**Total** *n*/ *n*(%) [95% CI]	**Intervention school 1 (rural)** *n*/ *n*(%) [95% CI]	**Intervention school 2 (urban)** *n*/*n*(%) [95% CI]	**Control school (urban)** *n*/*n*(%) [95% CI]
Number of year 9 and 10 pupils at participating schools	674	115	328	231
Number of parents/guardians who ‘opted out’ pupil	7 (1.0) [0.4%, 2.1%]	0 (0.0) [0%, 0%]	4 (1.2) [0.3%, 3.1%]	3 (1.3) [0.3%, 3.8%]
Number of pupils who consented to take part in the E4T feasibility study	481 (71.4) [67.8%, 74.8%]	90 (78.3) [69.6%, 85.4%]	233 (71.0) [65.8%, 75.9%]	158 (68.4) [62.0%, 74.3%]

^*^If a parent/guardian opted out, data for that pupil was removed from the data set, even if the pupil in question had consented to data collection

### Evaluate the appropriateness of the data collection procedures including acceptability to pupils and staff involved in implementing the intervention

Completion rates for E4T quantitative research measures in each of the schools pre- and post-intervention are shown in Table 3 below.

**Table 3:** Completion rates of data collection procedures in the E4T feasibility study

	**Control school** ***n* = 158 pupils** ***n* (%)** **[95% CI]**	**Intervention school 1 (rural)** ***n* = 90 pupils** ***n* (%)** **[95% CI]**	**Intervention school 2 (urban)** ***n* = 233 pupils** ***n* (%)** **[95% CI]**
**Questionnaires**			
Pre	95 (60.1) [52.0%, 67.8%]	68 (75.6) [65.4%, 84.0%]	133 (57.1) [50.5%, 63.5%]
Post	77 (48.7) [40.7%, 56.8%]	64 (71.1) [60.6%, 80.2%]	0 [0%, 0%]
**Canteen observations**^**a**^			
Pre	-	19 (21.1) [13.2%, 31.0%]	19 (8.2) [5.0%, 12.4%]
Post	-	16 (17.8) [10.5%, 27.3%]	13 (5.6) [3.0%, 9.4%]
**24-h recalls**			
Pre (weekday)	12 (7.6) [4.0%, 12.9%]	16 (17.8) [10.5%, 27.3%]	21 (9.0) and 21 (9.0) [5.7%, 13.5%]
Pre (weekend)	15 (9.5) [5.4%, 15.2%]	15 (16.7) [9.6%, 26.0%]	0 [0%, 0%]
Post (weekday)	15 (9.5) [5.4%, 15.2%]	14 (15.6) [8.8%, 24.7%]	18 (7.7) [4.6%, 11.9%]
Post (weekend)	18 (11.4) [6.9%, 17.4%]	0 [0%, 0%]	14 (6.0) [3.3%, 9.9%]

^a^Complete observations, i.e. both pre- and post-meal picture taken

#### Questionnaires

Two schools completed the questionnaires via the online survey platform (control and rural intervention school), while one chose paper completion (urban intervention school) owing to limited computer facilities. The questionnaire booklet took approximately 30 min to complete.

The urban intervention school was unable to arrange completion of the post-intervention questionnaires owing to other activities taking place in the school.

A small number of pre-questionnaires (*n* = 19; 6.4%) and post-questionnaires (*n* = 15; 10.6%) were only partially completed (i.e. questionnaire started but not finished). There was at least one question response or field missing in most pre-questionnaires (*n* = 236; 79.7%) and post-questionnaires (*n* = 118; 83.7%).

#### Assessing dietary behaviours

(i) Food purchasing data

Transaction histories for individual pupils covering the period of the intervention were successfully obtained for all schools.

(ii) Online completion of 24-h dietary recall

Organisation of online completion of 24-h recalls with one class in each school was challenging as they required the use of computer facilities and class time. The control school chose to ask pupils to complete the 24-h recalls on laptops during their lunch period to avoid disrupting class time. It was not possible to organise a weekend recall (on a Monday) with the selected class in the rural intervention school post intervention as it did not suit the timetable. Completion of the recalls with pupils required researcher supervision to allow queries to be answered, assist pupils with searches and try to avoid discrepancies between the search terms entered in INTAKE 24 and foods and beverages selected by pupils.

(iii) Completion of canteen observations

Two observations were completed at each intervention school canteen to capture food consumed; one before intervention commencement and one when the scheme was up and running at each school. Logistical challenges noted by researchers were that it was difficult to ensure pupils visited the photography table in the busy canteen environment before they took their seat to eat their food and after they had finished their food. This was partly owing to study staff restrictions; two researchers were conducting the observation protocol but did not have time to track all pupils and encourage them to visit and re-visit the photography table. Pupils chose their own seats in the canteen so tracking pupils who needed a post-meal photograph was also challenging. Stickers, given to pupils when queuing for the canteen, were used to identify pupils whose plates needed to be photographed (a numbered sticker for pupils’s blazer and tray). It would be ideal to do this in alphabetical order for tracking purposes, but this was not logistically possible in a short and busy lunch break. Some pupils misplaced stickers with their ID which made them more difficult to track and two pupils had to go to music practice as soon as they purchased their food.

Researchers also noted that both teachers and canteen staff mentioned that food choices served were healthier than usually served on other days and mentioned to researchers that they felt the need to make the food ‘look better’ as it was being photographed. With regard to reference portion photographs, one school was reluctant to ‘waste’ food for a reference photograph as the food could not be consumed after. The other school allowed researchers to photograph reference portions, but the food was served moments before pupils entered the canteen which meant it was challenging for photographs to be taken before pupil observations commenced.

#### School views about data collection (intervention schools)

Thirty-four pupils across the two intervention schools took part in five focus groups; *n* = 13 year 9 pupils; *n* = 21 year 10 pupils; *n* = 25 male, *n* = 9 female. Two interviews were conducted with school principals (one from each intervention school), two with canteen supervisors (one from each intervention school) and one with a teacher who championed the scheme. The main considerations for conducting the focus groups were similar to those for questionnaires: finding a suitable timetable slot to conduct focus groups and minimising class time that was missed. The logistical demands of organising data collection for research within a busy school environment were acknowledged in the interviews with principals.

‘What people forget is, you’re one of many things that impacts on the class, like you have sport, you could have the nurses and medicals, you could have other school trips, curricular trips etc. and they can all impact’ (Principal 1).

Interruption to normal class time was a concern and schools helped to minimise such disruption.

‘I don’t think it’s (data collection) had any impact really and if we’re careful when we plan, when you do your surveys then, you don’t annoy the teaching staff’ (Principal 1).

A suggested solution put forward by one principal to reduce the impact of data collection on classes in the future was to incorporate it into the school curriculum (e.g. 24-h dietary recalls could be completed as part of a Home Economics lesson plan).

‘instead of looking at a form class, maybe you would look at a home economics group or something, I don’t know, there’s another way’ ‘Part of their curriculum they do look at nutrition so that might be a natural element, I’m only thinking of that now, that would be a natural follow on’ (Principal 1).

Another point raised by both principals was that if they were to participate in a future scheme, they would delegate responsibility for the scheme and data collection to a member of teaching staff as their own time was so limited.

‘I think to get the commitment and continuity and to get things resolved quickly, you probably need the person closest on the ground cause the more…the further up, the further away, the less time the… So someone who has responsibility for health is the ideal person to kind of work with you from the beginning’ (Principal 2).

### Evaluate the acceptability of the E4T scheme to pupils and stakeholders involved in implementing the intervention

Findings are described below in terms of registration for the scheme, engagement with the scheme and overall acceptability.

#### Registration process

In readiness for the E4T scheme going live, pupils had to visit the website and register for the scheme (step 1). This sign-up procedure required pupils to use their school email and password to confirm their identity and complete registration (step 2). Overall, 79% of pupils completed step 1 and 55% completed step 2. In the rural school, 83% completed step 2, whereas in the urban school 45% completed the registration (Table 4). Some pupils, particularly within the urban school, had difficulty activating their accounts as they were either unfamiliar with their school email login details, or were unable to locate the account activation email which was automatically diverted to pupil’s junk mail folders by the school email server.

**Table 4:** Proportion of pupils registering for the E4T scheme

		**Total** ***n*/*n*(%)** **[95% CI]**	**Intervention School 1 (rural)** ***n*/*n*(%)** **[95% CI]**	**Intervention School 2 (urban)** ***n*/*n*(%)** **[95% CI]**
Number of consenting pupils at intervention schools who completed step 1^a^ of the sign up to the E4T scheme		256 (79.3) [74.4%, 83.6%]	79 (87.8%) [79.2%, 93.7%]	177 (76.0) [70.0%, 81.3%]
Number of consenting pupils at intervention schools who completed registration for the E4T scheme (step 2)^a^		179 (55.4) [49.8%, 60.9%]	75 (83.3) [74.0%, 90.4%]	104 (44.6) [38.1%, 51.3%]
Gender and year group of consenting pupils who completed registration for the E4T scheme:	Female	32 (17.9) [12.6%, 24.3%]	32 (42.7) [31.3%, 54.6%]	0 [0%, 0%]
	Male	147 (82.1) [75.7%, 87.4%]	43 (57.3) [45.4%, 68.7%]	104 (100.0) [96.5%, 100.0%]
	Year 9	111 (62.0) [54.5%, 69.2%]	47 (62.7) [50.7%, 73.6%]	64 (61.5) [51.5%, 70.9%]
	Year 10	68 (38.0) [30.9%, 45.5%]	28 (37.3) [26.4%, 49.3%]	40 (38.5) [29.1%, 48.5%]

^a^Step 1—Pupils visited the E4T website and registered for the E4T scheme (step 1). Step 2—Pupils use their school email and password to confirm their identity and complete registration for the E4T scheme

A number of pupils commented that the registration process on the E4T website was difficult The main issues reported included unfamiliarity with school email log in details, mis-entered details (i.e. discrepancies between information entered and information stored on pupils in the school canteen) and activation emails being diverted to the junk mail folder. Another problem raised during the focus group discussions was that pupils often forgot the usernames and passwords they set up to access their E4T account on the website.

‘It’s [registration] hard cause you had to go to the junk folder [to find email needed to validate your account]’ (M, FG4).

These registration difficulties caused disruption at the start of implementation, which then reduced the weeks that the scheme ran in schools.

‘It seemed like a lot because of the delay at the start. If that had happened cleanly, it would have all appeared much more spread out and calm and organised and in these steps, but that didn’t happen and therefore we had weeks of trying to get log-ins resolved and the emails back and forward, which kind of then meant that everything else seemed a wee bit squashed together’ (Principal 2).

All staff suggested having a pocket-sized card with the details they needed for registration would help the process.

#### Engagement

Throughout the intervention, there were 2855 sessions on the E4T website, 852 (30%) of these were from returning visitors. The biggest proportion of sessions (37.4%) were accessed during school hours. Most website sessions were accessed via a desktop computer (86.6%). Those who accessed the website using other devices mostly used Apple iPhone (56.7%), followed by Android phones (33.9%), Apple iPad (7.8%) and Apple iPod (1.6%). Per month of the scheme, website session numbers were: September (*n* = 691), October (*n* = 559), November (*n* = 907), December (*n* = 526), January (*n* = 202). The sections of the website that were visited most frequently (ordered from most to least page views) were rewards (i.e. rewards available), login (to personal account), website homepage, join in (register to E4T), my profile (pupil’s individual profiles), points (i.e. the points associated with each food/beverage) and news. A total of *n* = 35 out of 179 pupils (19.6%) individuals downloaded the E4T Android App. The E4T website was deemed informative, attractive and easy to use by pupils.

‘It was good, well laid out. If you had any queries like, you just go and it will tell you’ (F, FG2).

Throughout the scheme, 41 (23%) pupils who consented to take part in feasibility study and successfully registered for E4T scheme claimed a reward (Table 5). Of rewards claimed, 71% were claimed by pupils from the rural intervention school. A greater proportion of pupils from the rural school (36.6%) claimed rewards than pupils from the urban school (9.7%). In the co-ed rural school, 14 out of 43 (32.6%) males and 15 out of 32 (46.9%) females claimed rewards. The most commonly claimed type of reward was stationery (69 out of 85 rewards (81%)), followed by sports items or vouchers (6 out of 85 (7%) each) and electronic items (4 out of 85 (5%).

**Table 5:** Summary of rewards claimed during the E4T scheme^a^

	**Total** ***n* (%)** **[95% CI]**	**Intervention school 1 (rural); *n* (%)** **[95% CI]**	**Intervention school 2** **(urban); *n* (%)** **[95% CI]**
Number of rewards claimed	85	66 (78%) [67.3%, 86.0%]	19 (22%) [14.0%, 32.7%]
Number of pupils that claimed reward/s	41	29 (71%) [54.5%, 83.9%]	12 (29%) [16.1%, 45.5%]
Type of reward:			
Stationery (e.g. eraser)	69 (81%) [71.2%, 88.8%]	56 (85%) [73.9%, 92.5%]	13 (68%) [43.5%, 87.4%]
Sports (e.g. football)	6 (7%) [2.6%, 14.7%]	4 (6%) [1.7%, 14.8%]	2 (11%) [1.3%, 33.1%]
Electronic items (e.g. earphones)	4 (5%) [1.3%,11.6%]	3 (4.5%) [1.0%, 12.7%]	1 (5%) [0.1%, 26.0%]
Vouchers	6 (7%) [2.6%, 14.7%]	3 (4.5%) [1.0%, 12.7%]	3 (16%) [3.4%, 39.6%]

^a^Based on *n* = 179 pupils who consented to take part in feasibility study and successfully registered for E4T scheme

Discussion during the pupil focus groups indicated that once E4T was up and running, a number of pupils forgot about the scheme and became disengaged. Pupils suggested a number of methods to improve engagement, including personal reminders (e.g. frequent visits from researchers to school assemblies, and reminders from school teachers and canteen staff) and technology-based reminders (e.g. notif1ications/automated text messages displaying points to mobile phones, displaying information on the school website and screens in school hallways and via social media).

Another issue raised in relation to engagement was that pupils had limited opportunity during the school day to check the points they had earned. Pupils reported this was because time allocated for computer usage was for completing class work rather than personal use.

‘In school we are always told to get off the internet and do what we’re doing or do what we are meant to be doing’ (M, FG5).

The E4T app was only available for Android phones which limited usage, and there was limited discussion about it in focus groups but there was some indication that it was more convenient to use at home than the website, and that it was a quicker means of checking their E4T account than via the website.

‘It’s easier to go on like at home or anything’… ‘It was quicker’ (F, FG1).

School staff also suggested methods for improving pupil engagement in a future scheme. One suggestion put forward by a school principal was to offer group/class/ ‘house’-based rewards. Like pupils, they also suggested further promotion of the scheme via the school website and the mobile phone app.

Another suggestion given by the same canteen supervisor to increase pupil engagement with the scheme was to promote it via the school’s TV system (screens placed in the hallways and canteen) and website ‘Believe it or not the wee lads actually do sit and watch the tv…it’s on a loop but you’ll find them just sitting starting at it you know’ (Canteen supervisor 2).

‘…and also on the school website…cause even that way, you know some of the parents go on to ah, check the school holidays’ (Canteen supervisor 2).

Finally, a suggestion was put forward by a canteen supervisor to offer discount vouchers to use in the school canteen as a reward, which is something the school currently offers for good behaviour.

#### Acceptability of the E4T scheme

(i) The overall concept of E4T, branding and promotion

Overall, pupils were very receptive to the concept of receiving rewards for eating healthily in the school canteen, with only positive feedback given about the concept during focus group discussions. Pupils suggested that receiving rewards for eating healthily was motivational and fun and introduced a sense of competition. No harms or unintended consequences were reported.

‘It’s a fun way to not eat like burgers or chips and all’ (M, FG5).

‘Me and him were like having a competition for the most points!’ (M, FG4).

E4T branding (i.e. the study logo and study name) and promotional materials (i.e. posters and the study website) were well received by the majority of pupils. With regard to the study name, a number of pupils commented that it was ‘catchy’ and that it reflected the aim of the scheme ‘It means what it says’ (M, FG1).

Similarly, the study logo was described as ‘colourful’, ‘eye catching’ and ‘noticeable’.

(ii) Rewards

There was a high degree of acceptability for the (type/range/variety) rewards offered to pupils in E4T.

‘The wee prizes aren’t extortionate but you know for what they are, the kids love it’ (Teacher 1).

However, some pupils suggested that some of the rewards (particularly those with the highest monetary value) seemed unattainable during the time frame of the scheme.

‘Does it actually take about 900 years though to get a big prize?’ (M, FG2).

A number of pupils also mentioned a delay between claiming and receiving their rewards. Suggestions were put forward by pupils to make the reward retrieval process more efficient. For example, enabling pupils to collect rewards from their school office rather than waiting on teaching staff to distribute them or having them distributed in assembly, which two pupils described as embarrassing, particularly as they were retrieving small prizes. Some pupils also suggested that group/class-based rewards would be well received.

‘Mine was hectic to get’… ‘If we could like pick it up from reception, instead of like having to go to a teacher’ (M, FG4).

‘There could also be like, there’s an individual one and then there could be like a whole class points put together’ (M, FG5).

For the feasibility study, researchers undertook administration of rewards and delivered them to schools to be given out by principals. Both principals acknowledged the delay in their distribution of rewards.

‘And so the Eat4Treats has been sitting with me, maybe for 10 days and a few of them approached me’ (Principal 1).

(iii) Perceived benefits of the scheme—pupils

Pupils discussed some ways they felt the scheme raised their awareness of healthy eating and impacted on their dietary choices but some also acknowledged that they just continued to eat normally.

‘More about things that you wouldn’t have maybe thought was really healthy, they actually are healthy’ (F, FG2).

‘You could see that people were starting to do it. People were eating healthier’ (M, FG4).

‘I only get what I like’ (M, FG2).

(iv) Perceived benefits of the scheme and acceptability—staff perspective

School teaching staff were also positive about the concept of E4T and believed it fitted well with the curriculum and with the healthy eating policies already in existence within their schools.

‘Well like, we would consider ourselves a school that’s really trying to educate people for the future, so I think it fits in really well because a healthy body’s a healthy mind and someday then hopefully they will do better in class’ (Principal 1).

Canteen supervisors talked positively about the potential value of the E4T scheme and how it was received.

‘Great idea, the kids were just absolutely amazed’ (Canteen supervisor 1).

‘I think it would be a good idea to put it through all the schools’ (Canteen supervisor 2).

‘You know encourage them to eat a wee bit healthier, if they know they are going to get prizes for it, like a point system, you know especially in an all-boys school it is going to encourage them because they are very competitive’ (Canteen supervisor 2).

School staff mentioned that they felt pupils enjoyed the scheme, and the teacher interviewed noted that she thought it had some positive impacts on pupil’s dietary behaviour. Staff felt that pupils were more likely to eat meals than to snack and that some pupils who were entitled to free school meals were more likely to avail of them during the scheme.

‘My form class is in the ICT room in the mornings for our form class and they are all logging on, checking their points, checking out what food will allocate them more points, very competitive and definitely have enjoyed being involved. In fact are very, very, very disappointed that it will come to an end…’ (Teacher 1).

‘You know, they’re not just going up and picking cookies or something that’s handy, they are actually sitting down and having a meal’ (Teacher 1).

‘And also I’d have found that some of the pupils who would have missed lunch, even those who do get free school meals are using now their meal ticket to actually purchase their meal’ (Teacher 1).

Another benefit of E4T mentioned by school staff was that it was useful in terms of increasing discussion about healthy eating within the school.

‘You know and it’s great and it started a lot of conversation too about food’ (Teacher 1).

Other benefits mentioned by principals and teachers included that E4T stimulated positive competition between pupils and that it allowed the school to exert a certain level of influence over the school canteen via encouraging healthy food choice among pupils.

‘…I would hear some of the children talking and trying to get points, so a wee bit of competition between each other…’ (Principal 1).

‘We are keen that there are healthier options in the school canteen and we directly don’t have any control over what’s served, so that seemed like a good way to…obviously you know influence or have some discussions about what was offered there’ (Principal 2).

Both canteen supervisors suggested that they had noticed some positive changes in pupil’s purchasing behaviour including increased choice of healthier sides such as potatoes. However, there were mixed reports on whether vegetable purchases had increased, and one supervisor also acknowledged mixed levels of engagement from pupils with regard to the scheme.

‘We are going through more salad stuff, we are going through more fresh fruit, we are going through less chips, we are going through more the likes of potatoes, you know more vegetables we are going through so I think the kids are trying to bump up their prizes’ (Canteen supervisor 2).

### Evaluation of resources needed to implement the E4T scheme and collect outcome data

To prepare for implementation of the E4T scheme, a significant amount of work was required to modify till interfaces for appropriate collection of purchasing data and to develop the E4T website.

#### Modification of till interface

Although the cashless canteen technology was in operation in schools, it was not configured in a way that was compatible with implementing the rewards scheme or monitoring food purchasing. The research team worked an IT expert from the CCS provider, each school’s catering manager and the Regional Food in School co-ordinator to modify the till interfaces to capture purchasing data compatible with the reward point framework. This work required multiple visits to schools to discuss menus, current till layout and modification of the till layout, set up the modified till interface on the CCS and train canteen staff in the new till layouts. Overall, it took one school year to complete this till set-up work in the four recruited schools, which included a trial period of using the new till layout before collection of any baseline data.

Feedback from canteen staff operating the new till interface indicated that they were ‘wary at the start’ and there were some teething problems during initial implementation. For example, one canteen supervisor reported that it slowed the speed of service at the till at first; however, the other supervisor did not report this as being an issue.

‘Service was slower…you know like we were still putting them through the counters at our normal speed, not realising that they have an extra couple of buttons to hit per child’. (Canteen supervisor 2).

The supervisors also mentioned that reducing the habitual use of the ‘miscellaneous button’ and increasing the accuracy of button selection among canteen staff was challenging but they appreciated why it was important.

‘you know like we had a miscellaneous button which I know people don’t like. Yous don’t like it because if we put through miscellaneous yous don’t know what it is. But at the same time, the only reason why we need that is, say if a child comes in, you know we have to feed them no matter what, and if they’re coming in and they haven’t got enough money on their account’ (Canteen supervisor 2).

After a period of familiarisation, supervisors reported that canteen staff became acquainted with the layout, that it had benefits including improving staff awareness of the menu (e.g. what is included in meal deal combinations), and having a more organised and easy-to-follow till interface due to the colour coding of different food categories.

‘Now they’ve got used to it. I think at the beginning it was you know familiarising themselves with the tills. I had actually printed out the till screens for them’ (Canteen supervisor 2).

‘There we go, and it went well, went live with colour and everything and it went smooth for both staff and pupils, very well’ (Canteen supervisor 1).

Both supervisors reported that the support and training provided by the cashless canteen company and by researchers was valuable in helping staff to adjust to the new till interface and troubleshoot any teething problems.

‘The good thing is cause they do remote access so do you know if I had a problem I’d just phone them and they never had to come out I explained the problem over the phone, and they just sat in their office and…’ (Canteen supervisor 2).

‘I think they (cashless canteen company) and you (research team) have worked really well with us’ (Canteen supervisor 1).

Staff appreciated having familiarisation time with the new till layouts before the scheme formally started.

‘We had it for a while so that they could get used to putting the itemised stuff through. You know that that was good. That was like having like a two months training for them’ (Canteen supervisor 2).

When asked about potential improvements for the scheme, one canteen supervisor put forward the idea of introducing barcodes on packaged items such as sandwiches that could be scanned at the till to record more detailed information about the food item, although the cost of such a service was acknowledged.

‘The only other way round that would be barcode it, and scanning them through…but then that there’s going to be a big expense and sure no one has any money to go down and do all these new labels and buy these scanners’ (Canteen supervisor 2).

#### Website operation

Connection of the E4T website to school systems was the main technical challenge with website operation. The E4T website was set-up to pull data daily from the CCS regarding food purchased and use this to calculate a points total for each pupil. To link this canteen purchasing data to the individual pupil registered on the E4T website required a unique identifier to link the pupil on the E4T website with the pupil in the CCS. This created some challenges with security firewalls which blocked the transfer of data from the CCS to the website and had to be resolved through discussions with the school’s IT team and the Department of Education. To ensure pupils on the two systems were linked correctly, the system set-up also required pupil information, such as class lists, held by the canteen to match school year group lists; in one school, pupil information had not been updated on the canteen system and had to be corrected which required administrative support from the school.

‘what you were doing was quite an ambitious project because you’re coming into a very different computer system and then you are trying to link the meal system up with [the school server/network]’ (Principal 2).

In the other intervention school, the transfer of purchasing information from the school canteen to the E4T website was initially blocked by the schools server, and the principal suggested that she felt ill equipped to solve the problem when approached by the research team.

‘And I see an email [from the study researchers] about checking systems and I’m going, I don’t know how to check systems, I don’t do that ahh help!’ (Principal 2).

The website sign-up process that caused some difficulty for school staff with regard to the scheme was the registration process. The principals and teacher mentioned that this was problematic not only in terms of the time required and the impact on classes, but also owing to the various issues encountered as mentioned previously. Principals mentioned that giving pupils a physical reminder of their details required to register would help to solve the problems associated with registration such as a small card with the details needed, or asking pupils to write down their account login details in a safe place when registering could help.

‘it’s that process of registering. That just knocked the whole thing on the head. If it had been simple, I think the whole thing might have had a much bigger impact’ (Principal 2).

#### Staff employment for data collection

In terms of staff resources for data collection for the feasibility study, one full-time post-doctoral researcher was employed to implement the intervention and conduct the data collection. However, two staff members were needed for some elements of data collection, notably the completion of 24-h recalls, canteen observations and focus groups.

## Discussion

### Summary

The paper describes the development and feasibility testing of a reward scheme based on food purchasing behaviour in cashless canteens in secondary schools. It set out to explore the feasibility of recruiting schools and implementing this novel intervention in the school canteen setting and to evaluate the acceptability of both the intervention itself and research approaches that could potentially be used to assess its effectiveness.

The E4T scheme was successfully implemented and results of the feasibility study indicate that the intervention shows promise based on high acceptability for the scheme among pupils and staff as well as feedback describing perceived benefits. There was a high level of interest in the scheme and all feedback received about the concept from pupils and staff was positive. Schools felt it fitted well with the curriculum and their school healthy eating policies. Pupils liked the branding and found the website easy to use and informative. There was high acceptability for the type and variety of rewards offered which had been chosen based on pupil consultation. Pupils felt the scheme was motivational and fun and introduced a sense of competition which was also echoed by the school and canteen staff. Pupils and staff felt the scheme raised awareness about healthy eating, promoted discussions between pupils about food choices and had some positive impacts on behaviour such as feeling pupils were more likely to choose meals rather than snack-type option, choosing healthier side options and more fruit and also encouraging uptake of free school meals. However, it was also acknowledged that engagement from pupils was mixed.

Feedback from pupils and staff, as well as researcher observations from the study log, indicated some key recommendations to improve user experience in the future which are discussed below along with implementation and data collection considerations.

### Technical challenges implementing

The main challenges to implementing E4T were technical in nature, particularly in relation to preparing the CCS for capturing good-quality purchasing data and the pupil registration process.

A high percentage of eligible pupils were interested in taking part in the E4T scheme as illustrated by attempted sign-ups via the E4T website; however, difficulties with the sign-up process resulted in a much lower registration completion rate. This could be overcome in future work by using an alternative means of validating pupil’s accounts, for example through text message or personal email accounts. There may also be merit in providing pupils with any personal information required for the registration process (e.g. full name, class, school email address) to speed up the registration process. Technology use in schools is constantly increasing and evolving and the COVID-19 pandemic has meant that pupils are now using their school email and other school-based technology on a regular basis compared to when the E4T feasibility study was conducted, thus it is likely that the registration issues we encountered would be less problematic in the future. Indeed, some use of personal mobile phones is now permitted in schools, for example during ‘form’ or ‘registration’ classes or for specific purposes in other lessons. These advances in use of technology for education purposes would also aid the implementation of a scheme such as E4T as pupils would be able to register and engage with the scheme more easily.

A substantial proportion of the preparatory work prior to the launch of E4T focused on making changes within the school canteen to enable the point system to function. This study showed that it is feasible to implement such a system in cashless canteens successfully. With regard to the process, it took considerable 18 months to set-up in CCS. In NI, schools operate their own menus and hence the preparatory work to modify till interfaces for the E4T scheme had to be done on a school-by-school basis. In terms of a future trial, time required by all stakeholders (researchers, CCS suppliers and canteen staff) to implement E4T could be reduced considerably if, for example, it was launched within certain regions in the UK which operate standardised school menus. This would enable one standard till interface to be implemented across a number of schools. Within a future trial, it would also be useful to consider whether pupil information systems (i.e. pupil names, classes) within the school and canteen are linked as this was not the case in one E4T intervention school and resulted in extra administration on behalf of office staff.

### Engagement and rewards

Engagement with the scheme was variable with many pupils claiming rewards but some pupils indicating they forgot about the scheme. The research team’s original plan to remind pupils that the scheme was running and encourage them to engage via their school email addresses was not successful as pupils did not use their email often, and the research staff complement for the study limited the number of times the researchers could visit each school. As discussed above, use of technology and pupil’s familiarity with their email and login details has increased following the COVID-19 pandemic which is likely to benefit future interventions. A future trial should consider sending automated reminders to pupils via mobile phone/app and should account for regular research visits to school assemblies to raise the profile of the scheme. A future trial could also be further integrated into the school curriculum (e.g. part of a health or home economics class) at the school agreement which could increase pupil exposure to and engagement with the scheme. Findings from pupil focus groups also suggest engagement may have been low due to some pupils feeling that high-end rewards were unattainable. A future trial may benefit from giving pupils examples of daily meal choices, which could enable retrieval of the pupil’s reward of choice. The thresholds for the high-end rewards were designed to require pupils to make the best choices on most days of the week for the duration of the intervention. Thresholds could also be varied over time to stimulate engagement, for example, using strategies employed in retail settings such as point promotions and leveraging behavioural economics methodologies such as discrete choice experiments to optimise the reward approach [26].

The process of reward retrieval in E4T was also described as a barrier by some pupils. A future trial should order rewards in bulk to ensure faster transfer of rewards from researchers to the schools, and a process by which pupils can collect their rewards at a certain location/time each week should be established. Another potential method as suggested by some pupils and a principal to increase engagement in a future trial would be to promote competition among pupils, including, for example, through introducing an element of group/class rewards.

### Collecting research data

With regard to research processes, although we successfully recruited four schools, one school, a control school, withdrew before the intervention commenced. This decision was made by the school principal and unfortunately came after a significant amount of preparatory work had been done to reconfigure the till interface in the school canteen to allow surveillance of purchasing data. Even though the principal had been fully informed about the research at the outset, they decided to withdraw when it came to baseline data collection which required teacher assistance. The use of a signed memorandum of understanding alongside a token of appreciation for the school that is proportionate to the level of involvement of the principal, teachers and other staff may help maximise retention of schools in trials [27]. The research team noted that in the three schools who completed the research, there was a high level of connectivity between the school principal and the school canteen, i.e. the canteen was viewed as an integral and vital part of the school rather than as a separate entity and this may be important for the success of research involving the school canteen as the principal is the overall gatekeeper. This connectivity between the principal and the school canteen was not evident in the school that withdrew from the study.

The nature of the study, burden on schools and desire to ensure as many pupils as possible participated were all considered when developing the research study consent process. Very few parents chose to opt-out their children and this approach has been discussed before as a valuable way of recruiting a more representative sample of pupils [28]. Pupil consent was ‘opt-in’ and was moderately successful as consent form return rate varied between schools. Informal feedback from one teacher suggested that the consent form may have been portrayed as too official or formal which may have led to privacy concerns and been off-putting for pupils. A future study should consider PPI engagement to further refine the layout of the consent form, perhaps using imagery to describe the study rather than just text and a researcher attending class to talk about study and answer questions may also boost consent.

When working with schools, co-operation and assistance from teachers is generally essential to oversee consent form completion and the burden of this needs to be taken into account by researchers and ethics committees when developing and approving consent processes. The consent process should be appropriate for the nature of the research being undertaken but should consider how to maximise participation and therefore representativeness of the sample.

The main observation with regard to data collection in relation to the study was around the logistics and staff time required to collect detailed dietary data and other outcomes in the school setting. While data collection was possible, it was time intensive and created a number of logistical challenges owing to the busyness of the school environment. For questionnaires, online completion is preferable in terms of encouraging completion of all fields but was not always possible owing to limited access to IT facilities. Access to computer suites was also required for 24-h recalls, with a double period required for 24-h recall. We requested that one of the two 24-h recall sessions was completed on a Monday to collect data on a typical weekend day. This was not always possible as timetabling limited class availability. One way to overcome this is to provide tablets to pupils for completion of online questionnaires; however, this would add significantly to research equipment budgets, although it is likely that developments in IT availability within schools, particularly post COVID-19, will see more widespread availability of tablets within schools. Overall, discussion with school staff indicated it can be challenging to organise time to complete study measures such as questionnaires and time taken away from other aspects of their school work or school day needs to be considered; however, schools were willing to try to find workarounds for this such as using form class time or other health-related classes to complete.

Researcher presence at time of questionnaire completion would likely reduce the extent of missing data or partial questionnaire completeness but this requires a higher research staff complement. Pre-questionnaire completion was similar across schools; however, it was apparent that partial post-questionnaire completion was higher at the rural intervention school than the control school. Both completed these online and researchers proposed that this difference may be attributable to the intervention questionnaire having a greater number of items than the control school questionnaire as additional items were included to evaluate the study. For these reasons, careful consideration needs to be given to the minimum data collection that is required to evaluate effectiveness in school-based trials. It is better to collect a smaller amount of high-quality data than a large amount of poor-quality data.

In order to evaluate the impact of an intervention that aims to influence and reward food purchasing behaviour, it is desirable to evaluate what food is served, purchased and eaten as pupils may purchase foods for their point value but not actually eat them. Collecting information on what is served was possible via school menus provided by the canteen staff; however, this menu can deviate from what is planned, for example, if there are supply issues or to use up stock towards the end of school terms. In terms of food purchased, the CCS produces date- and time-stamped transaction histories for every pupil who buys food in the canteen and so has huge potential as an objective means of data monitoring. However, as discussed above, CCS are not necessarily optimally configured for these purposes when set up in schools. In this study, a significant amount of work was required to configure the CCSs to capture sufficient detail on foods/beverages purchased. Even with better potential for data capture at the till, there is still a reliance on catering staff accurately keying in items purchased, interviews with canteen staff indicated that they understood the need to do this; however, actual practice may be variable between staff, between school and even day-to-day depending on pupil traffic in the canteen. This has previously been highlighted as a limitation of CCS as a means of collecting dietary data in schools [29]. Canteen observations are a way of examining food eaten and food wastage but school canteens are high footfall, quick throughput areas, with most schools operating a first and second lunch sitting. Measuring food waste on an individual pupil basis requires multiple staff observers and a lot of pre-planning to optimise the process and, based on this feasibility study, such observations would be ambitious to do in a large-scale trial.

Overall, when designing trials in the school setting, it is important to be realistic about outcome data collection, consider core outcomes needed to address the key research questions and, if more in-depth data is desired, consider doing this in a sub-sample of schools, after collection of key outcome data. Studies require realistic levels of research staff and appropriate recognition for the school in terms of the level of assistance and support required to collect the research outcomes. Outcome data collection has to be acceptable to schools and participants but also realistic from a research staff complement perspective and often compromises have to be made based on research budgets available. Establishing a working partnership with schools from the planning stage of research will help to set the stage for effective recruitment, successful protocol execution and intervention implementation [30].

### Strengths and weaknesses

Strengths of this study include that it is the only existing study to report on the feasibility and acceptability of a novel food-based reward scheme among adolescent school pupils. This feasibility study focused on ‘extended’ schools which are schools serving areas of highest social disadvantage and it demonstrates the potential of such an approach to reach all children, including those entitled to free school meals, without risk of stigmatisation. Research targeting nutritional inequalities is needed to help combat socially differentiated patterns in health. The scheme itself focused on making better choices and the concept of balance and variety; these concepts underpin healthy eating guidelines and reflect evaluation skills young people will need to navigate food choice decisions in the real world.

Limitations include the small number of schools included; although this may be deemed suitable for feasibility purposes, it is difficult to generalise the findings. In some cases, convenience samples of pupils were used to test certain research measures such as the 24-h recall, or take part in the focus groups, which may also reduce the generalisability of the findings. Finally, the qualitative interviews were carried out by CR who had established a rapport with the principals and canteen staff, which may have encouraged a more positive response. However, the fact that all parties freely discussed potential improvements to E4T would suggest this was unlikely. Finally, data on cost-effectiveness was not collected in the feasibility study and this would be an important component of any larger trial.

### Future work

Based on the findings from this feasibility study, it was clear that the E4T intervention was well received by pupils and could be implemented in secondary school canteens operating a CCS. The concept of a reward scheme to encourage pupils to make better choices in the school canteen, or purchase food from the canteen in preference to other alternatives, shows promise. Progressing this work to an effectiveness trial would require careful consideration of the preparatory work required to set-up such a scheme in schools, mainly time required to set up the till interfaces in school canteens and for staff to become familiar with the layout. This would require investment of dedicated resource to achieve this in the required number of schools in a reasonable timeframe. This intervention would be more straightforward to implement in areas that operate standardised menus across schools as till interfaces would not have to be tailored to each individual school. It is also possible that some schools already have their CCS set-up to gather good-quality purchasing data which would reduce the preparatory work required to implement the scheme. Resource would also be required to run the scheme; administration work for the feasibility study was undertaken by the research team. If rolled out on a wider basis, an administrative assistant would be required to ensure smooth operation of the scheme, trouble-shoot, keep the website live and up-to-date and ensure timely delivery of rewards to pupils.

Some refinements would be needed to encourage sustained engagement with the scheme, for example, by implementing automated reminders sent to pupils or presentations in school assemblies. Finally, some technical aspects would need to be optimised around registration logistics (i.e. problems activating E4T account due to unfamiliarity with school email addresses and passwords), transfer of data from school canteen to E4T website, linking pupil information with canteen data. This would require appropriate support from school regulatory bodies such as governmental education departments as well as some support from schools to ensure school pupil lists match those on CCSs.

A full trial, with an embedded pilot, could be used to ensure these issues were adequately addressed before proceeding. Future trials should also include consider a sustainable model of support for wider implementation should effectiveness and cost-effectiveness be shown.

Trialling an intervention such as this alongside other potentially synergistic interventions should be considered to maximise impact. Existing research indicates that many factors influence pupils’ choice to eat in the school canteen and the choices they make when they are there. Length of queues, canteen aesthetics (the physical environment and presentation of the food itself), hygiene, value for money and more limited food choice for pupils assigned to second lunch sitting are all important. Systematic reviews of interventions aimed at improving dietary behaviour in secondary school pupils provide evidence that multicomponent interventions, i.e. those combining education and changes to the environment, are more successful than environmental or educational approaches alone [31].

## Conclusions

In conclusion, the E4T reward-based scheme was successfully implemented in this non-randomised controlled, feasibility study as a result of collaboration between schools, school canteens and cashless canteen providers working with a multidisciplinary research team. It was acceptable to both the pupils and staff involved in implementing the scheme. The concept caught the attention of pupils and an increased awareness of food choices and competition with peers was reported as a result of engagement with the scheme. The findings suggest a number of areas for intervention refinement that would need to be considered in the design of a larger trial, particularly with regard to resources required for its implementation and ways to optimise pupil engagement. The outcome assessment approaches should also be refined to prioritise collection of a minimum dataset required for evaluation of effectiveness. This work highlights the potential of a reward-based intervention focusing on food choice in the school setting to reach young people across the socio-economic spectrum and also demonstrates the value of conducting feasibility studies, particularly for novel, technology-focused interventions such as E4T.

### Supplementary Information

**Additional file 1: Table S1.** Behaviour Change Techniques (BCTs) incorporated into the Eat4Treats scheme.. Description of Behaviour Change Techniques used in Eat4Treats and how these BCTs were incorporated into the intervention.

**Additional file 2: Figure S1.** Eat4Treats Website Homepage (Pre-Registration). **Figure S2.** Eat4Treats Website ‘Join in’ section. **Figure S3.** Eat4Treats Website Homepage (Registered and Logged in). **Figure S4.** Eat4Treats Website ‘Points’ section. **Figure S5.** Eat4Treats Website ‘Rewards’ section. **Figure S6.** Eat4Treats Website ‘Information’ section. **Figure S7.** Eat4Treats Website ‘My Profile’ section. Images from the Eat4Treats website sections.

**Additional file 3: Figure S8.** Example of till interface at one school captured after till modifications. **Figure S9.** Example of food purchasing till outputs at one school before and after modifications were made to the till interface. Examples of school canteen till interfaces and food purchasing till outputs before and after modifications were made to improve data capture.

**Additional file 4: Table S2.** E4T scheme points framework including rationale. Description of the points framework developed for the E4T scheme including rationale for points allocated.

**Additional file 5: Table S3.** Sample questions from focus group topic guide exploring pupil experiences of taking part in Eat4Treats. **Table S4.** Sample questions from interview topic guides exploring school staff experiences of taking part in Eat4Treats. Sample questions from focus group and interview topic guides exploring experiences of taking part in the study.

## Data Availability

The datasets used and analysed during the current study are available from the corresponding author on reasonable request.

## Abbreviations

BCT: Behaviour change technique
CCS: Cashless canteen system
E4T: Eat4Treats (Intervention)
FG: Focus group
F: Female
M: Male
