# Supplementary material for: The feasibility and acceptability of a rewards system based on food purchasing behaviour in secondary school cashless canteens: the Eat4Treats (E4T) cluster feasibility, non-randomised, controlled intervention study
Source: Pilot Feasibility Stud. 2024 Jan 9;10:4. doi: 10.1186/s40814-023-01436-6 (PMC10775569; doi:10.1186/s40814-023-01436-6)

Additional file 2

Figure S1: Eat4Treats Website Homepage (Pre-Registration)

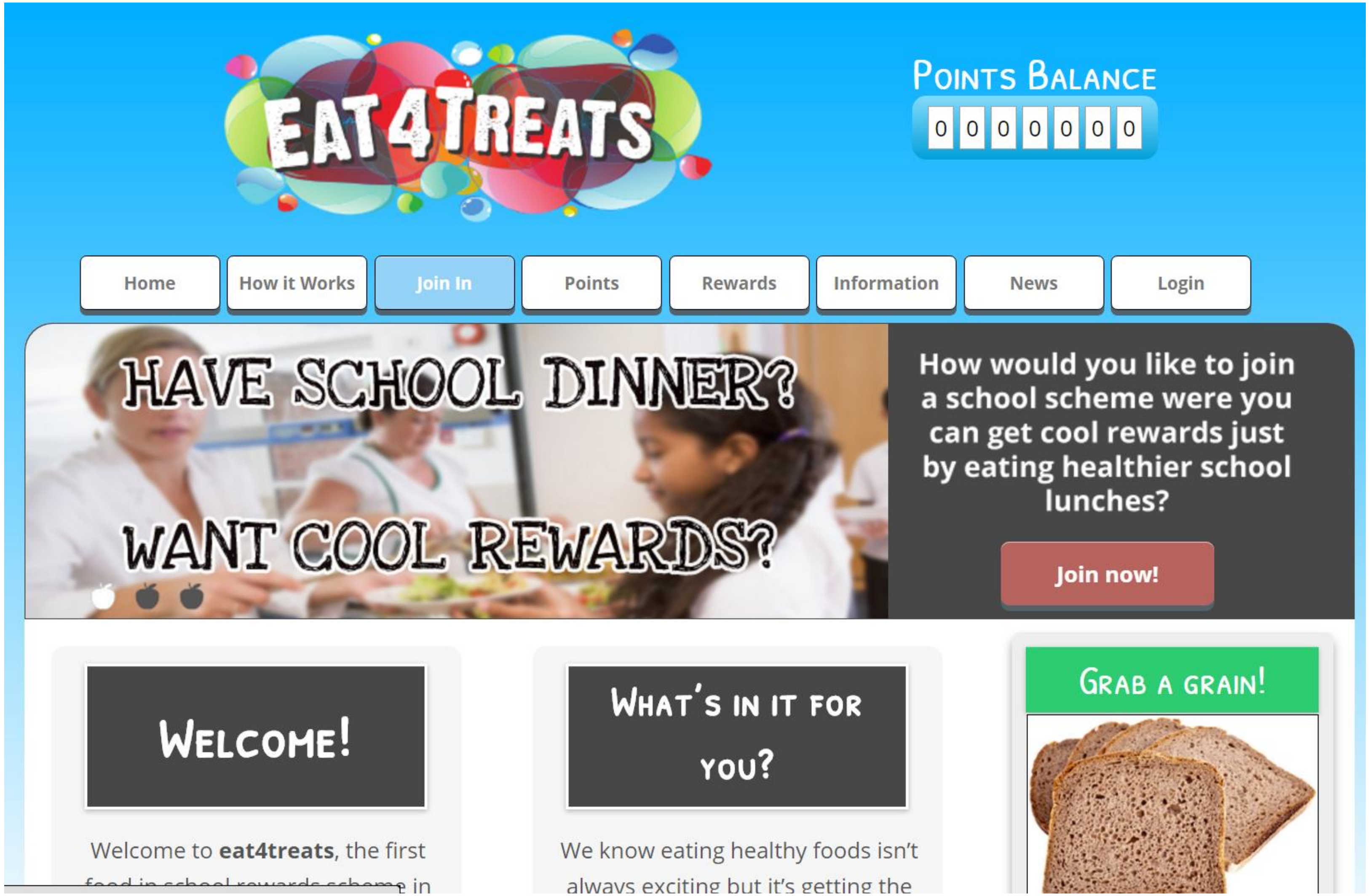

Figure S2: Eat4Treats Website ‘Join in’ section (Registration page)

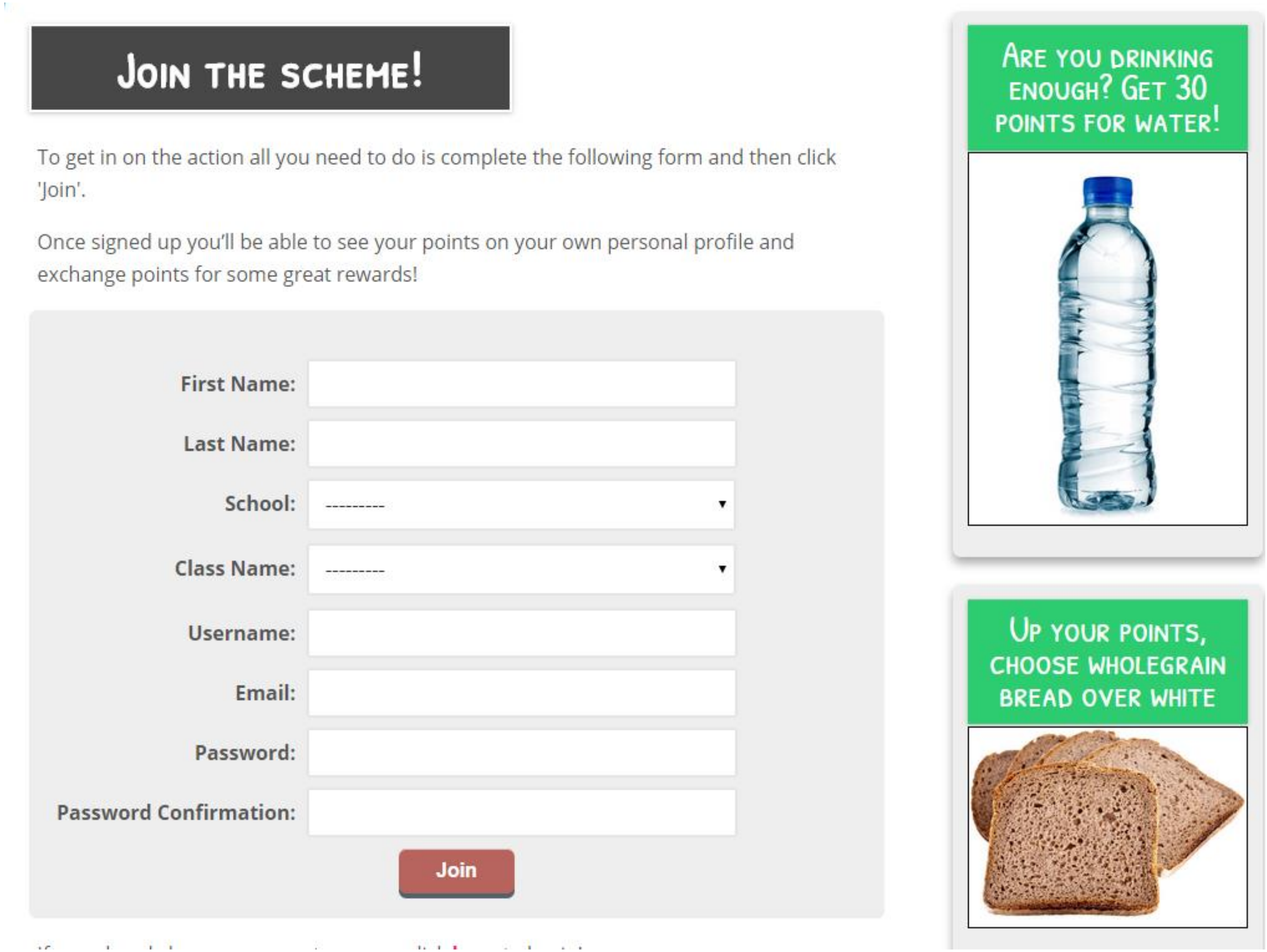

Figure S3: Eat4Treats Website Homepage (Registered and logged-in)

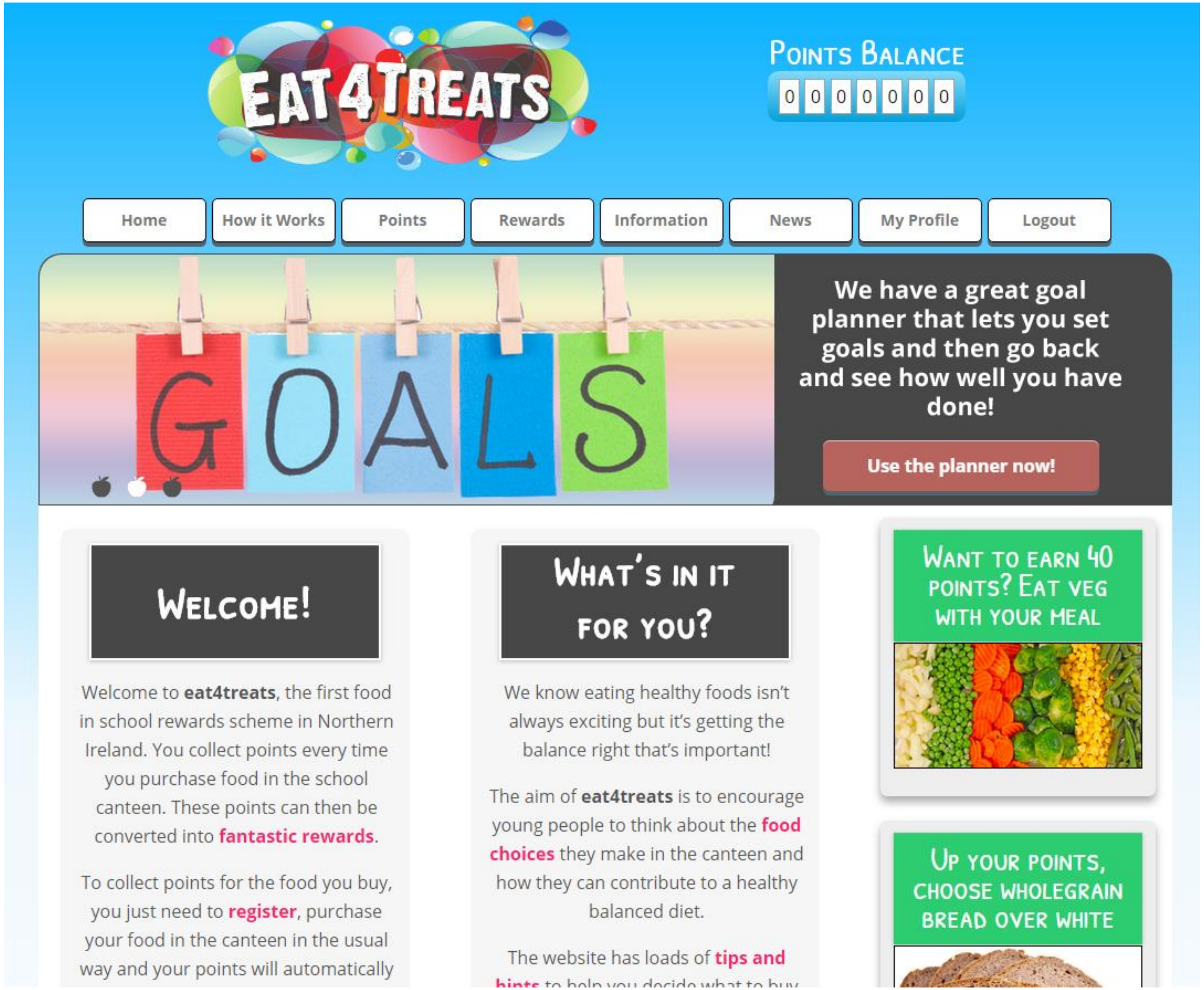

Figure S4: Eat4Treats Website 'Points' section (Outlining number of points awarded for choosing different foods)

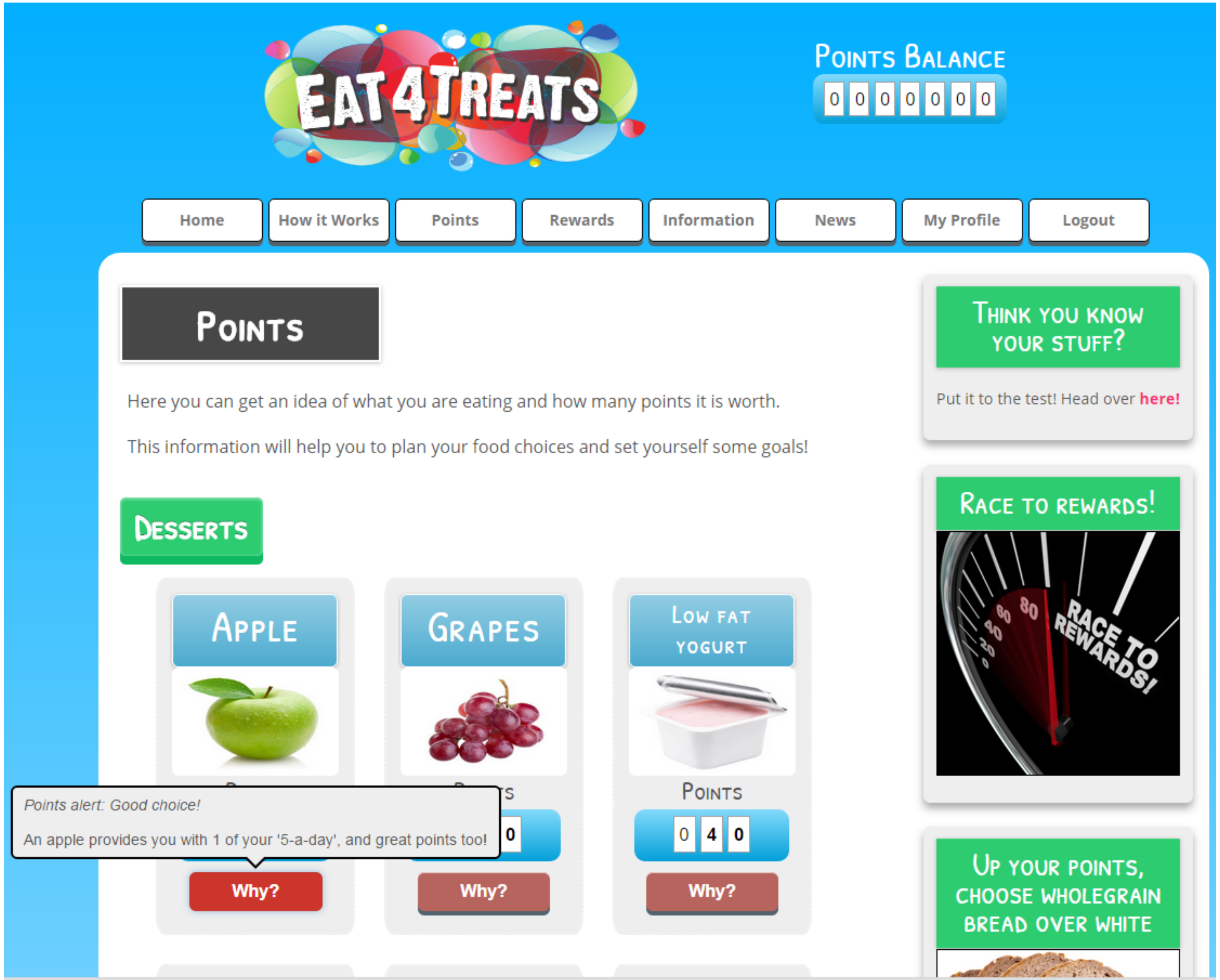

Figure S5: Eat4Treats Website 'Rewards' section (Outlining rewards available and points needed to claim rewards)

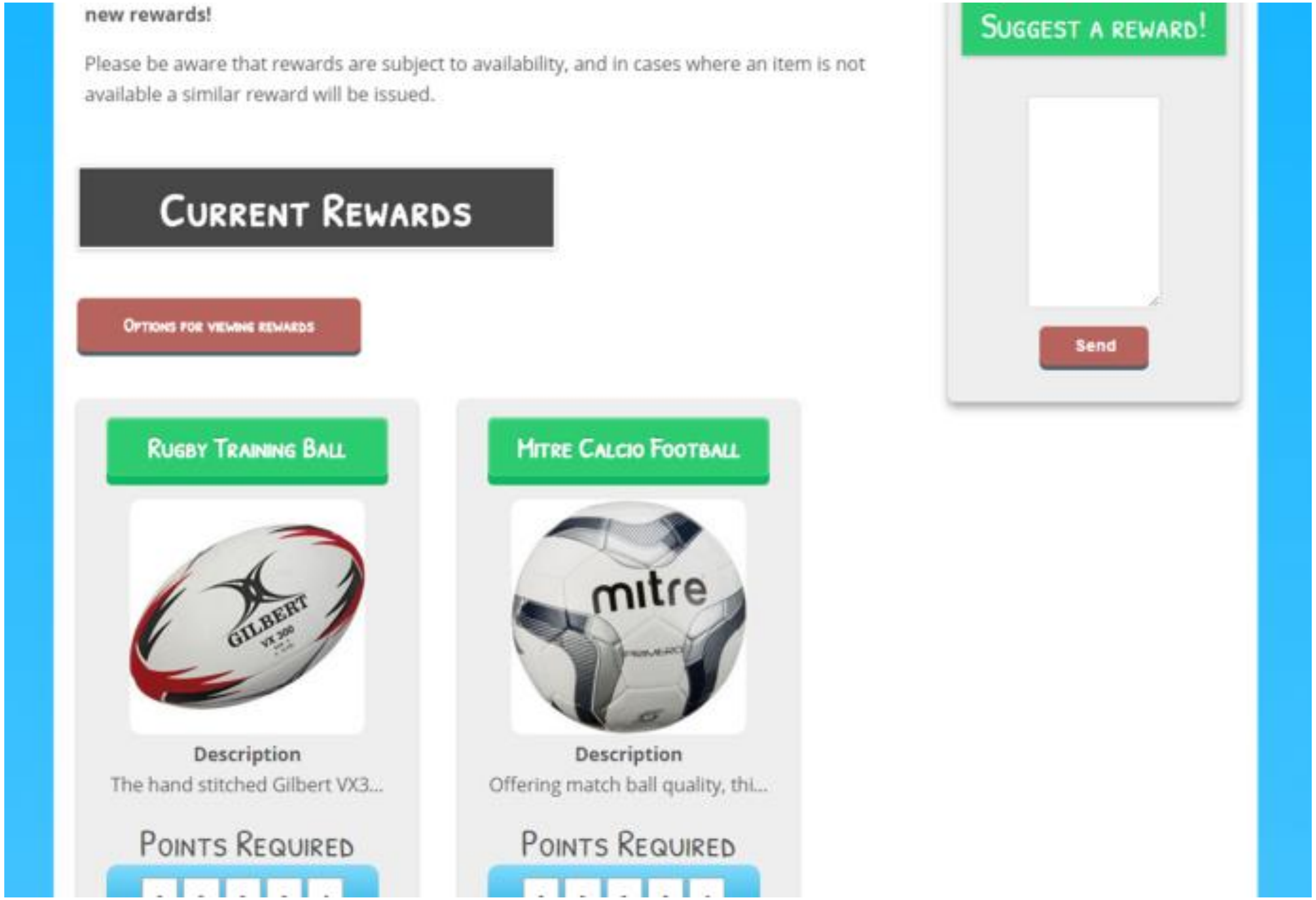

Figure S6: Eat4Treats Website ‘Information’ section (Nutrition Education)

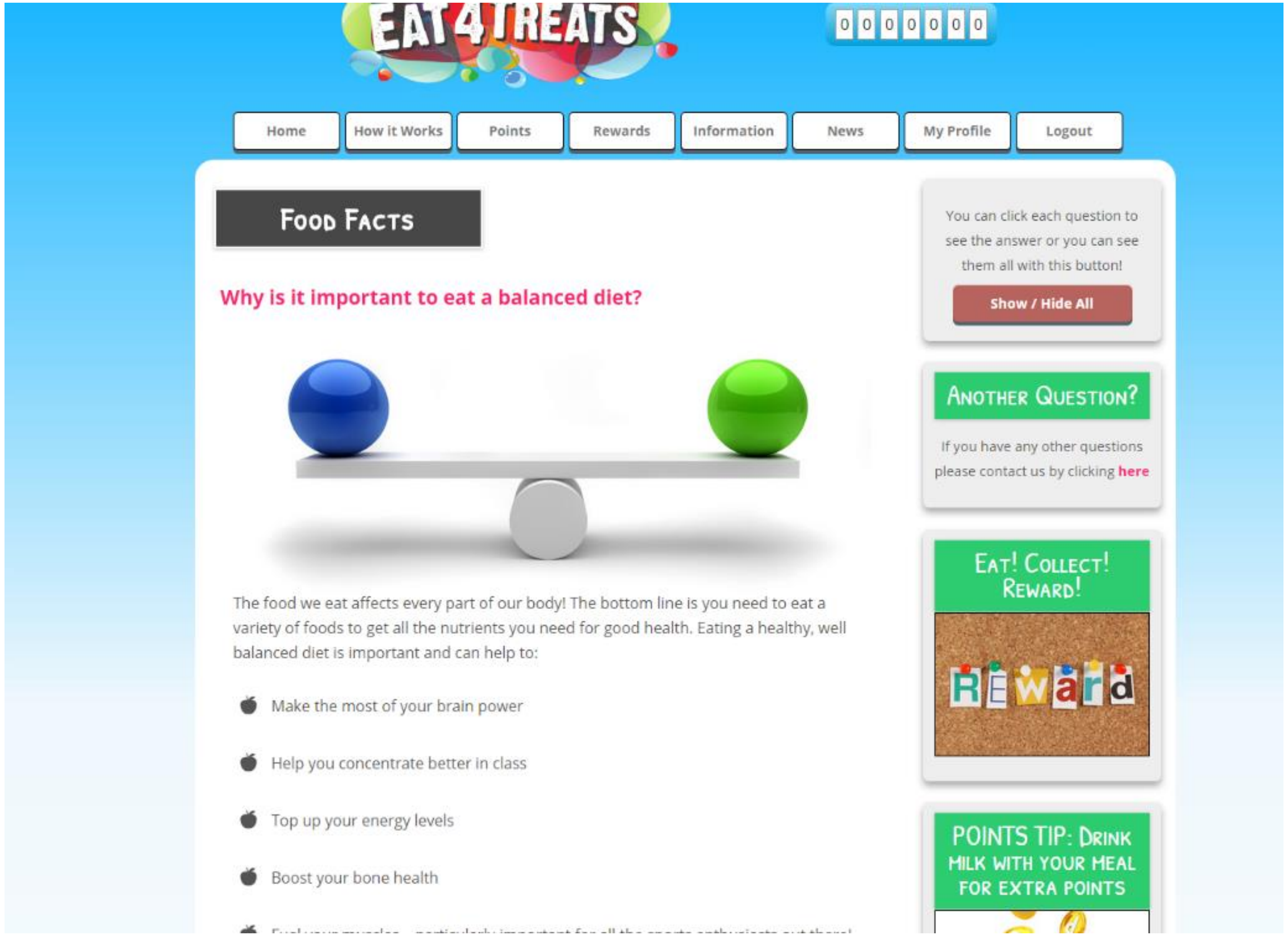

Figure S7: Eat4Treats Website 'My Profile' section (Displaying points balance and record of rewards claimed)

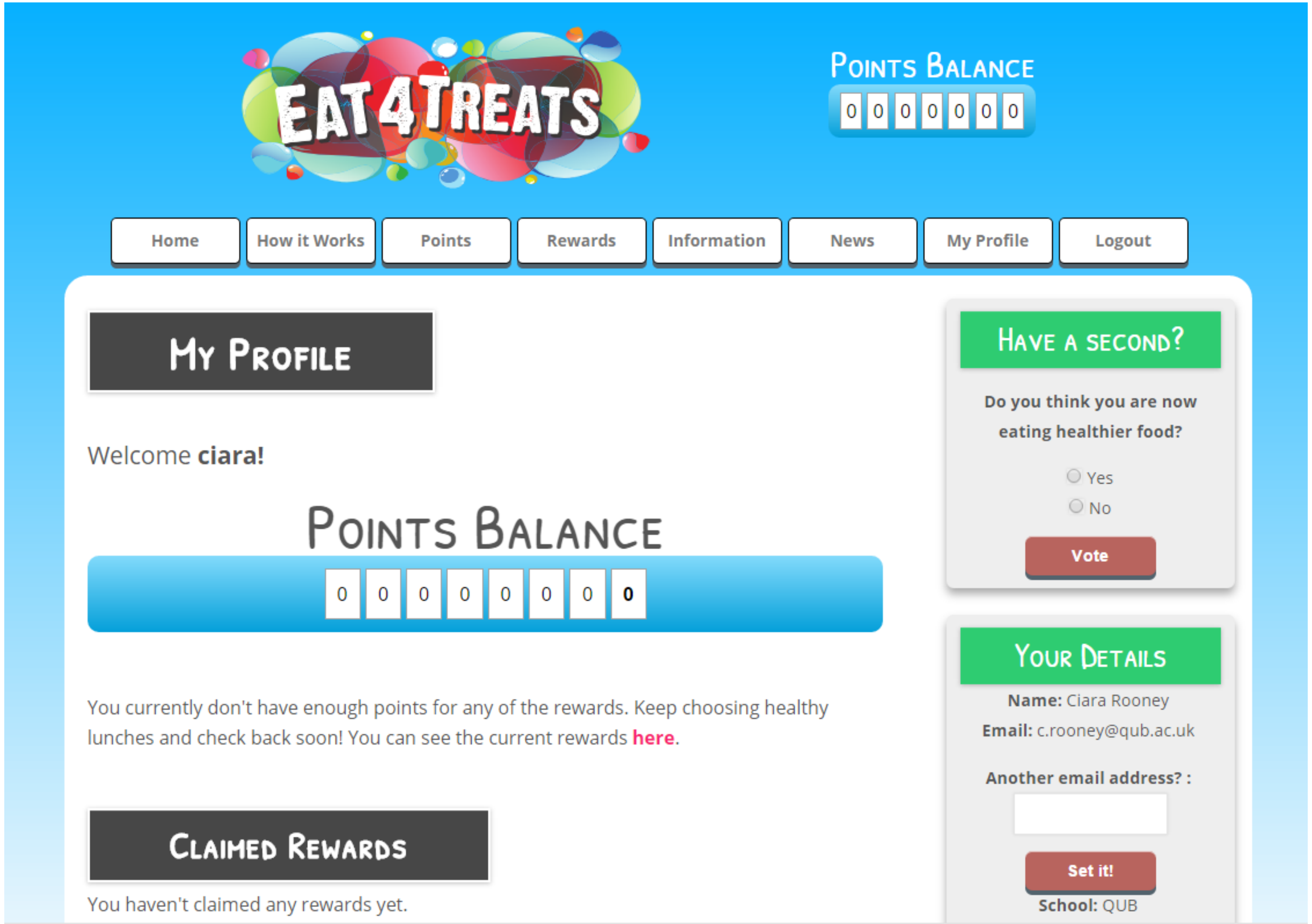

Supplement: Supplementary file 2 — Additional file 2: Figure S1. Eat4Treats Website Homepage (Pre-Registration). Figure S2. Eat4Treats Website ‘Join in’ section. Figure S3. Eat4Treats Website Homepage (Registered and Logged in). Figure S4. Eat4Treats Website ‘Points’ section. Figure S5. Eat4Treats Website ‘Rewards’ section. Figure S6. Eat4Treats Website ‘Information’ section. Figure S7. Eat4Treats Website ‘My Profile’ section. Images from the Eat4Treats website sections. [file 40814_2023_1436_MOESM2_ESM.pdf]
