# Supplementary material for: The feasibility and acceptability of a rewards system based on food purchasing behaviour in secondary school cashless canteens: the Eat4Treats (E4T) cluster feasibility, non-randomised, controlled intervention study
Source: Pilot Feasibility Stud. 2024 Jan 9;10:4. doi: 10.1186/s40814-023-01436-6 (PMC10775569; doi:10.1186/s40814-023-01436-6)
Supplement: Supplementary file 3 — Additional file 3: Figure S8. Example of till interface at one school captured after till modifications. Figure S9. Example of food purchasing till outputs at one school before and after modifications were made to the till interface. Examples of school canteen till interfaces and food purchasing till outputs before and after modifications were made to improve data capture. [file 40814_2023_1436_MOESM3_ESM.pdf]

Additional file 3

Figure S8: Example till interface at one school captured after till modifications

A. Screen 1: Lunch Main Meals

Steak burger with roll (£1.60)		Chips (£1.30)	Baked beans (£0.50)	Rice pudding with two fruits (£0.65)	Fresh fruit salad (£0.65)	Still water 330ml (£0.55)
Fish (£1.45)		Creamed / boiled potato (£0.40)	Peas (£0.00)	Jam sponge (£0.65)	Apples (£0.25)	Tea / Coffee / Cappucino (£0.65)
Donor kebab + pitta bread (£1.60)		Baked potato (not filled) (£0.70)	Tossed salad (£0.00)	Custard (£0.65)	Bananas (£0.30)	Hot chocolate (£0.65)
Sausages x2 (£1.20)		Baked potato (with cheese & baked beans) (£1.30)	Pasta salad (£0.00)	Jelly, fruit and custard (£0.65)	Grapes (£0.50)	Milk semi 250mls (£0.30)
Pasta bolognaise (£1.80)		Boiled rice (£1.30)	Potato salad (£0.00)	Flakemeal biscuit (£0.40)	Oranges (£0.30)	Milkshake (£0.55)
Hot dog (£1.10)		Curry sauce (£0.50)		Yoghurt low fat (£0.50)		Fruice pure 200mls (£0.60)
Chicken curry with rice & naan bread (£1.80)		Gravy (£0.20)				Fruice juice drink 250mls (£0.65)
Chicken curry with chips & naan bread (£1.80)	Halal £1.80 meal (£1.80)					
Chicken curry 50:50 (£1.80)	Halal £2.50 meal (£2.50)					STAFF (£3.00)

C. Screen 3: Break

Toast – white (£0.25)	Pizza finger (£0.65)	Yoghurt (£0.50)	Still water 330ml (£0.55)	Butter portion (£0.10)	
Toast – wholemeal/ 50:50 (£0.25)	Digestive biscuits (£0.35)	Fresh fruit salad (£0.65)	Tea / Coffee / Cappucino (£0.65)	Flora portion (£0.10)	
Plain scone (£0.45)	Date fudge/krispie (£0.50)	Apples (£0.25)	Hot chocolate (£0.65)	Sauce/vinegar portion (£0.10)	
Cherry scone (£0.45)	Flakemeal biscuit (£0.40)	Bananas (£0.30)	Milk semi 250mls (£0.30)	Dairylea/Cheese spread portion (£0.25)	
Wheaten bread	Shortbread biscuit (£0.40)	Grapes (£0.50)	Milkshake (£0.55)		
	Cookie (£0.60)	Orange (£0.30)	Fruice pure 200mls (£0.60)		
			Fruice juice drink 250mls (£0.65)		

B. Screen 2: Lunch Salad Bar

White bd sandwich (no salad) (£1.90)	Baguette – white (no salad) (£1.90)	Soup homemade (£0.70)	Cheese spread portion (£0.25)	Yoghurt (low fat) (£0.50)	Still water 330ml (£0.55)	Wholemeal bread (£0.25)	Butter portion (£0.10)
White bd sandwich (with salad) (£1.90)	Baguette – white (with salad) (£1.90)	Soup with white roll (£1.00)	Digestive biscuits (£0.35)	Fresh fruit salad (£0.65)	Tea / Coffee / Cappucino (£0.65)	Plain scone (£0.45)	Flora portion (£0.10)
Brown/wholemeal/ 50:50 bd sandwich (no salad) (£1.90)	Panini - white – (no salad) (£1.90)	Soup with wholemeal roll/ 50:50 (£1.00)	Wholemeal / flakemeal biscuit (£0.40)	Apples (£0.25)	Hot chocolate (£0.65)	Cherry scone (£0.45)	
Brown/wholemeal/50:50 bd sandwich (with salad) (£1.90)	Panini – white (with salad) (£1.90)	Irish stew (£1.80)	Fruit based traybake / biscuit (£0.50)	Bananas (£0.30)	Milk semi 250mls (£0.30)		
Bap – white (no salad) (£1.90)	Tortilla wrap – white (no salad) (£1.90)	Salad box (meat, tuna) (£1.90)	Plain cookie / Chocolate cookie (£0.60)	Grapes (£0.50)	Milkshake (£0.55)		
Bap – white (with salad) (£1.90)	Tortilla wrap – white (with salad) (£1.90)	Salad box (vegetarian) (£1.45)		Oranges (£0.30)	Fruice pure 200mls (£0.60)		
					Fruice juice drink 250mls (£0.65)		

Figure S9: Example food purchasing till outputs at one school before and after till modifications were made to improve data capture

PRE TILL MODIFICATION

Pupil	Day	Time	Food purchased
4	5	BREAK	N/A
4	5	LUNCH	MEAL OF THE DAY
5	1	BREAK	N/A
5	1	LUNCH	N/A
5	2	BREAK	N/A
5	2	LUNCH	MEAL OF THE DAY
5	3	BREAK	N/A
5	3	LUNCH	PASTA/RICE POTS
5	4	BREAK	N/A
5	4	LUNCH	MISCELLANEOUS, TRAY BAKES
5	5	BREAK	N/A
5	5	LUNCH	2PASTA/RICE POTS
5	6	BREAK	N/A
5	6	LUNCH	MEAL OF THE DAY, FLAKEMEAL BISCUIT
5	7	BREAK	N/A
5	7	LUNCH	DIGESTIVE BISCUIT
5	8	BREAK	N/A

POST TILL MODIFICATION

Pupil	Day	Time	Food purchased
1	1	LUNCH	CHICKEN CURRY & RICE
1	1	LUNCH	FRUIT SALAD
1	1	LUNCH	BUTTER PORTION
1	1	LUNCH	PLAIN/CHERRY SCONE
2	1	BREAK	MUFFIN
2	1	BREAK	BUTTER PORTION
2	1	LUNCH	BAKED POTATO- NO FILL
2	1	LUNCH	BAKED BEANS
2	1	LUNCH	CHEDDAR CHEESE
3	1	BREAK	FRUICE PURE 200ML
3	1	BREAK	TOAST WHITE BREAD
3	1	LUNCH	COOKIE
3	1	LUNCH	PASTA POT
3	1	LUNCH	PASTA TOMATO
3	1	LUNCH	COOKIE
4	1	LUNCH	RICE POT
4	1	LUNCH	CURRY SAUCE
4	1	LUNCH	MUFFIN
4	1	LUNCH	COOKIE
4	1	LUNCH	FLAV WATER STILL
