# Supplementary material for: The feasibility and acceptability of a rewards system based on food purchasing behaviour in secondary school cashless canteens: the Eat4Treats (E4T) cluster feasibility, non-randomised, controlled intervention study
Source: Pilot Feasibility Stud. 2024 Jan 9;10:4. doi: 10.1186/s40814-023-01436-6 (PMC10775569; doi:10.1186/s40814-023-01436-6)
Supplement: Supplementary file 4 — Additional file 4: Table S2. E4T scheme points framework including rationale. Description of the points framework developed for the E4T scheme including rationale for points allocated. [file 40814_2023_1436_MOESM4_ESM.pdf]

Table S2: E4T scheme points framework including rationale

Foods and beverages were broadly categorised into ‘main meals’, ‘sides’, ‘sandwiches’, ‘drinks’ and ‘desserts’. Points were assigned for each category based on a number of factors: current food purchasing habits, UK healthy eating guidelines[1] and the relative healthiness of choices within each category. Every item on the menu was assigned a points value in-line with the ethos of encouraging balance and variety and encouraging pupils to have a complete meal rather than more snack-like options.

[1]NHS (UK National Health Service) Live Well, Eat Well. https://www.nhs.uk/live-well/eat-well/.

MAIN MEALS

Ranking (highest points to lowest points)	Points	Rationale
‘Balanced’ dishes e.g.<br>Spaghetti/pasta Bolognese<br>Vegetable curry & rice<br>Chicken pie (potato top)<br>Chicken curry with rice & naan bread<br>Chicken & vegetable stir fry with noodles<br>Salad box with meat/ vegetarian<br>Savoury fish pie<br>Chicken jambalaya with rice<br>Irish stew/beef stew	100	These options are assigned the highest points to encourage pupils to eat balanced meals at lunch (i.e., meals that consist of meat, starchy carbohydrate, vegetables & in some cases dairy). Preliminary analysis of purchasing data showed that such meals, with the exception of the meal deal and chicken curry, were not currently popular choices among pupils in one of the participating schools.
Less healthy versions of the above dishes<br>e.g., Chicken curry with chips & naan bread<br>Chicken curry (half rice, half chips); pastry topped pies, creamy pasta dishes<br><br>Meat on its own (with no sauces), e.g.:<br>Roast beef/chicken/turkey<br>Pork chop<br>Braised steak<br>Fillet of fish<br>BBQ chicken drumsticks	60	Scored lower to encourage pupils to choose healthier meals (those in the category above) which are mostly lower in fat than the options in this category.<br><br>Meat on its own scored in this category to allow for the addition of sides to make a balanced meal.
Processed meat dishes, e.g.<br>Sausage & bean hotpot<br>Ham & potato bake<br>Quiche – cheese & ham (all quiche)	50	Dietary guidelines recommend lower intake of processed meats.
Pizzas (any variety)	20	Preliminary examination of purchasing data showed high consumption of pizza in participating schools – lower points allocated to encourage consumption of healthier, more balanced options.

Processed meats on their own (no side), e.g.<br>- Processed meats / meat dishes e.g., bacon, sausage, ham, chicken burger, hot dog, beefburger**	10	See above.

SIDES

Ranking (highest to lowest)	Points	Rationale
Vegetables/ starchy carbohydrate sides (e.g. all potatoes, pasta, noodles, brown rice*)	40	Dietary advice recommends that fruit and vegetables and starchy carbohydrates make up the majority of daily diet – hence these foods have been awarded the highest points value for sides.
White rice	30	*Where brown/wholegrain rice is sold it will earn greater points than white rice due to higher nutritional value (fibre).
Oven chips/wedges	5	Chips/wedges are worth less points than potatoes due to a) nutritional value and b) because preliminary examination of purchasing data showed high consumption of chips in comparison to other sides in the canteen.
Mayonnaise based salads/ pasta or potato salad (if these can be distinguished)	5	Higher in fat so lower points. In cases where it is not possible to distinguish type of salad, pupils will be awarded points for plain salad.
Sauces (e.g., curry sauce, gravy, ketchup)	1	Sauces earn a single point due to fat, salt and sugar content and lack of nutritional value.

SANDWICHES

Ranking (highest to lowest)	Points	Rationale
Wholemeal/brown/50:50 bread sandwich with salad	100	Higher in fibre than white bread, addition of salad (contribution to 5-a-day).
Wholemeal/brown/50:50 bread sandwich without salad	60	Higher in fibre than white bread, but no salad hence less points.
White bread sandwich/panini with salad	50	Lower points due to white bread but has salad.
White bread sandwich /panini without salad	40	Lowest points – white bread, no salad.

DRINKS

Ranking (highest to lowest)	Points	Rationale
Water and Milk	30	Water and milk are preferred drinks. Milk consumption is important amongst this age group in relation to meeting calcium requirements, but preliminary examination of purchasing data showed consumption is low.
Milkshakes (no added sugar)	20	Milkshake (without added sugar) has been awarded more points than fruit juice as the latter is high in free sugars. Based on preliminary examination of purchasing data, fruit juice is already currently a very popular choice amongst pupils in comparison to milkshakes (and whole fruit).
Fruit juice (counts towards 1 of 5 a day)	10	See above. Fruit juice ranked higher than the drinks below based on the fact that it provides 1 of pupils 5-a-day.
Water based juices<br><br>Milkshakes (with added sugar)<br><br>Tea/ coffee/ drinking chocolate	2	These choices were awarded the least points based on sugar content and/or lack of nutritional benefit.

DESSERTS

Ranking (highest to lowest)	Points	Rationale
Desserts with added fruit	30	These options tend to be lower in fat and added sugar and provide some micronutrients due to fruit.
All other desserts/ biscuits	5	
